# NIR-activated catechol-functionalized nanodiamond nanofibers for accelerating on-demand MRSA and *E. coli* biofilm eradication

**DOI:** 10.1186/s13036-024-00469-6

**Published:** 2025-02-05

**Authors:** Hyeonseo Park, Tejal V. Patil, Jieun Lee, Hojin Kim, Seong-Jun Cho, Ki-Taek Lim

**Affiliations:** 1 Department of Biosystems Engineering, Kangwon National University, Chuncheon, Gangwon-do 24341 Republic of Korea; 2 Interdisciplinary Program in Smart Agriculture, Kangwon National University, Chuncheon, Gangwon-do 24341 Republic of Korea; 3 Institute of Forest Science, Kangwon National University, Chuncheon, Gangwon-do 24341 Republic of Korea; 4 Department of Food Science and Biotechnology, Kangwon National University, Chuncheon, 24341 Republic of Korea

**Keywords:** ND@PDA, NIR, Photothermal, ROS, Antibacterial, Biofilm, Cell adhesion

## Abstract

The rise of antibiotic resistance has made bacterial infections a persistent global health issue. In particular, extracellular polymeric substances (EPS) secreted by bacteria limit the effectiveness of conventional antibiotics, making biofilm removal challenging. To address this, we created ND@PDA nanoparticles by coating the surface of nanodiamonds (ND) with polydopamine (PDA). These nanoparticles were then integrated into polyvinyl alcohol to fabricate PVA/ND@PDA nanofiber scaffolds, resulting in an innovative platform with enhanced photothermal, antibacterial and antibiofilm properties. Upon exposure to near-infrared (NIR) light, the scaffolds exhibited a significant photothermal activity, oxidative stress and effectively damaging key bacterial components, such as biofilm, bacterial membranes, and proteins. Additionally, the catechol groups in PDA provided strong cell adhesion and high biocompatibility on the nanofiber surface. Our research proposes a platform that not only effectively addresses antibiotic-resistant infections but also contributes to advancements in wound healing therapies by enabling controlled antibacterial action with minimal toxicity.

**Supplementary Information:**

The online version contains supplementary material available at 10.1186/s13036-024-00469-6.

## Introduction

Bacterial infections pose a significant risk to many people worldwide each year. While antibiotics can be used to combat these infections, prolonged exposure to antibiotics can lead to bacterial resistance [1, 2]. Resistant bacteria secrete extracellular polymeric substances (EPS), which restrict the penetration of antibacterial agents into biofilms, making their removal challenging [3]. Therefore, there is a need for new platforms with high antibacterial activity and biocompatibility.

Electrospun nanofiber scaffolds, with their small fiber diameter, high porosity, and high surface area-to-volume ratio, excel at adsorbing bacteria [4]. Additionally, they can maintain appropriate moisture levels at wound sites, inhibiting bacterial growth and promoting wound healing. Without any treatment, simply irradiating with NIR can increase the ambient temperature to a level that exhibits certain antibacterial effects, but when combined with materials such as nanomaterials, this effect is further enhanced [5–7]. Furthermore, nanofibers containing photoactive materials can convert light energy into heat energy upon NIR (near-infrared) stimulation, generating reactive oxygen species (ROS) through a photothermal effect. ROS can oxidize key cellular components such as the plasma membrane, proteins, and organelles, effectively eliminating bacterial strains. This approach does not induce antibacterial resistance and allows for precise control and consistent treatment with minimal toxicity and side effects [8–10].

Nanodiamonds (ND) have excellent mechanical properties, a high surface area, and tunable surface structures. They possess drug delivery capabilities and are non-toxic, making them ideal for biomedical applications. Additionally, the carbon in ND contributes to overall thermal conductivity, increasing heat transfer efficiency, and their stability under light exposure makes them effective for photothermal treatment [11–14]. However, several obstacles have hindered the application of ND. A major issue is the strong agglomeration of individual particles due to their high surface energy. This leads to the formation of covalently bonded agglomerates with larger and wider size distributions. Consequently, achieving consistent and controlled drug delivery in therapeutic applications may be challenging, necessitating some form of surface modification [15, 16]. To overcome this limitation, we coated polydopamine (PDA) as a surface modification agent.

**Scheme 1:**
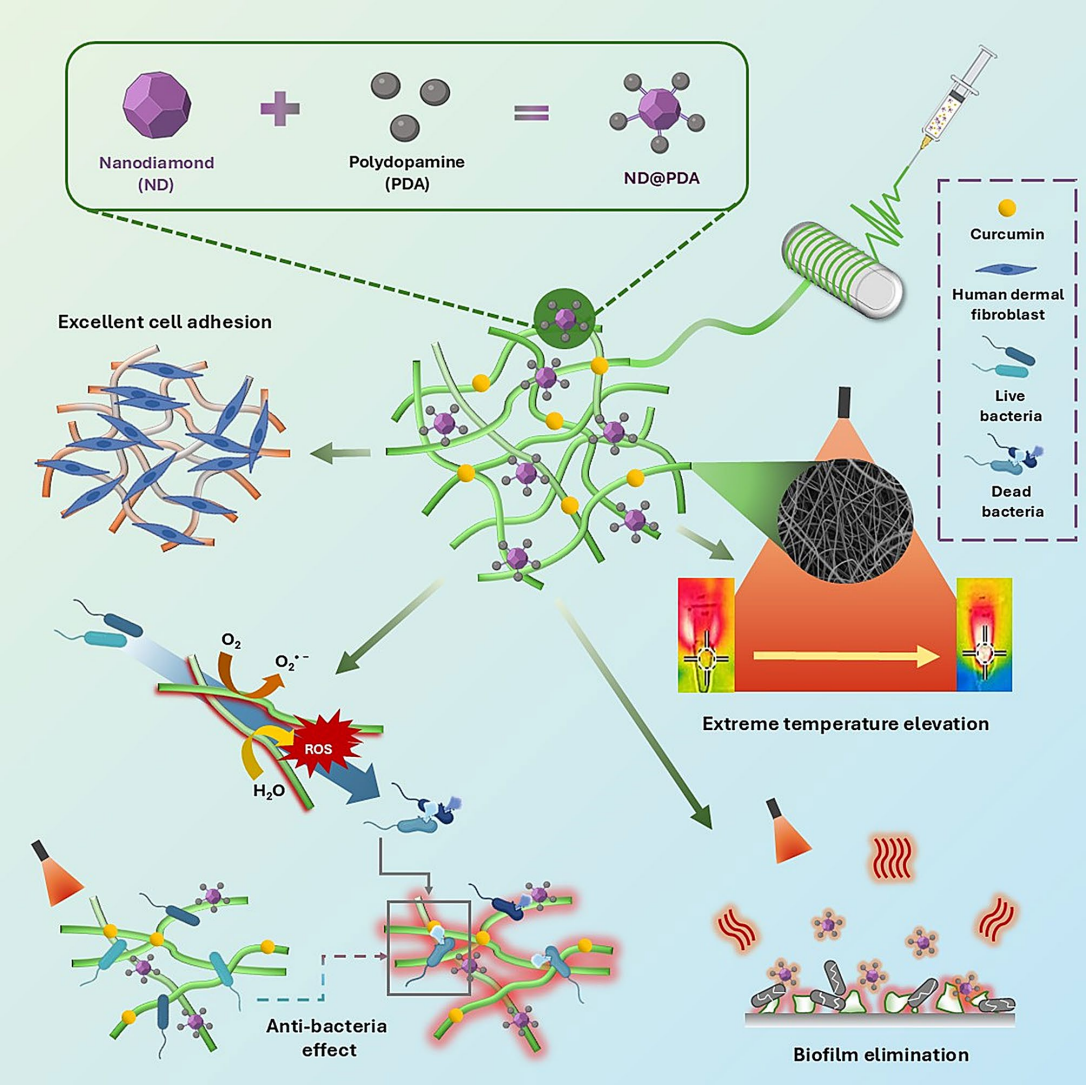
Synthesis of PNP nanofibers and their effects on temperature change, antibacterial, biofilm removal under NIR irradiation, and cell adhesion

Dopamine (DA), when exposed to an alkaline solution, undergoes self-polymerization to form a PDA coating that can modify material surfaces [17]. Dopamine is rich in catechol and amine functional groups that impart strong adhesion affinity to various substrates, inspired by marine mussel adhesive proteins. DA functions as an eco-friendly reducing agent, enhancing the biocompatibility and biodegradability of composite materials. It also possesses optical properties, making it suitable for photothermal therapy [17–19]. Our research combines these materials to create a platform that effectively kills bacteria on demand, and considering the biocompatibility of our samples, it can be a potential candidate for wound healing. To fabricate electrospun scaffolds, we synthesized ND@PDA nanoparticles with superior photothermal properties and investigated their antibacterial mechanisms under NIR exposure. Through this, we aim to provide new insights into wound treatment with enhanced antibacterial effects. The overview of our study in illustrated in Scheme 1.

## Experiment section

### Materials and reagents

Unless otherwise specified, we utilized all the compounds in their original form. Ammonium hydroxide (ammonia water), acetone, and polyvinyl alcohol (PVA, mol. Wt. of 22,000 g/mol) were purchased from Daejung Chemicals (Republic of Korea). Wako Chemicals (Republic of Korea) provided the hydrochloric acid (HCl). Diamond (nanopowder, < 10 nm particle size), dopamine hydrochloride (DA), ethanol, and curcumin were purchased from Sigma-Aldrich (USA). Glutaraldehyde (50% wt, certified) was supplied from Fisher Scientific (Ottawa, Canada). Difco™ Nutrient Agar, Difco™ Nutrient Broth, Difco™ Tryptic Soy Broth, and BBL™ Mannitol Salt Agar were purchased from Becton, Dickinson, and Company (USA). SYTO9 and propidium iodide were obtained from Invitrogen, Seoul, Republic of Korea. Crystal violet was purchased from the Tokyo Chemical Industry (Tokyo, Japan). WST-8 kit obtained from Cellrix^®^, MediFab Co., Ltd., Seoul, Republic of Korea. Human dermal fibroblasts (HDFs) and Methicillin-resistant *Staphylococcus aureus* (ATCC BAA-41) were purchased from America Type Cell Culture (ATCC) (Manassas, Virginia, USA). We obtained *Escherichia coli* (KCTC-2593) from the Korean Collection of Type Cultures (KCTC) located in Jeon-buk, Republic of Korea.

### Preparation of ND@PDA

Fabrication of ND@PDA nanoparticles was performed using the previously reported method [20]. In brief, 120 mg of DA was completely dissolved in 10 mL of deionized water, and 60 mg of diamond was added. Subsequently, the above solution was injected into a mixture containing 50 mL of ethanol, 90 mL of distilled water, and 1.5 mL of ammonia solution (28 wt% aqueous solution) and stirred for 24 h at 300 rpm. The color of the solution gradually changed from brown to black. The obtained ND@PDA was collected by centrifugation at 4000 rpm for 25 min and washed thrice with distilled water. The final wash was performed using deionized water, and the fabricated ND@PDA was dried at 40 °C for 2 days.

### Preparation of electrospun nanofiber

This study prepared different concentrations of PVA/ND@PDA (PNP) using 0.5%, 1%, and 2% w/v ND@PDA (Figure S1). Polyvinyl alcohol with an average molecular weight of 22,000 g/mol was gradually added under stirring conditions at 90 °C and 50 rpm to prepare a 15% (wt%) PVA solution. Next, appropriate amounts of 0.5%, 1%, and 2% ND@PDA dispersions were obtained by sonicating them in distilled water. In parallel, 15% PVA was prepared by adding PVA granules in distilled water at 90 °C with continuous stirring (50 rpm). ND@PDA dispersions were then added to the 15% PVA solution and stirred at 200 rpm for 3 h to ensure a homogeneous mixture. PNP nanofibers were obtained using an electrospinning machine (NanoNC Co., Ltd., Seoul, Republic of Korea). The collector used was plate-type, and aluminum foil was wrapped around it to facilitate easy removal of the obtained nanofibers. The PNP solution was loaded into a 10 cm^3^ syringe and a 21G needle was used. The polymer was pumped at a constant flow rate of 2.0 mL/h with a voltage setting of 15.9 kV/cm. The fabricated nanofibers were named PVA, PNP-0.5, PNP-1, and PNP-2, depending on the amount of ND@PDA dispersion added. The produced nanofibers were dried at room temperature for one day. For PNP-2/Curcumin (PNPC) nanofiber fabrication, 1% curcumin was added to the PNP-2 solution and stirred at 150 rpm overnight at 90 °C to obtain a homogeneous mixture. The obtained solution was electrospun under the same conditions as mentioned above. The cross-linking of PVA-based nanofibers was conducted by exposing the nanofibers to the vapor of a mixture of acetone, HCl, and glutaraldehyde for 15–30 min [21]. Finally, the crosslinked nanofibers were washed with distilled water and acetone to completely remove the crosslinking agents, followed by drying at room temperature.

### Characterization of ND@PDA and nanofibers

The morphology of ND and ND@PDA, as well as the nanofibers, was evaluated using Field Emission Scanning Electron Microscopy (FE-SEM, JSM-7900 F, JEOL, Japan). Additionally, the structural characteristics of ND and ND@PDA were assessed through Field Emission Electron Microscopy (FE-TEM, JEM-2100 F). The functionalization of organic functional groups in the samples was investigated using Fourier transform infrared spectroscopy (FTIR) from Thermo Scientific in the Republic of Korea. FTIR spectra were collected in the mid-infrared region (4000 –500 cm^-1^). The elemental composition of ND and ND@PDA was confirmed by EDS mapping analysis. X-ray diffraction patterns (XRD, Panalytical, X’pert-pro MPD, Netherlands) were recorded using Cu Kα radiation with a scan rate of 5 °C/min over the range of 5–90°. To confirm the elemental composition and bonding energies, additional analysis was achieved using X-ray photoelectron spectroscopy (XPS, K Alpha^+^, ThermoFisher, USA).

The characterization of the nanofibers was evaluated by SEM, FT-IR, and XRD following the parameters mentioned above. The thermal stability was investigated using a Thermal Analysis system (TGA, TA Instruments, SDT Q600, USA), where the samples were analyzed under a nitrogen atmosphere with a heating rate of 10 °C/min from 30 to 700 °C. The mechanical properties of the produced nanofibers, including PVA, PNP-0.5, PNP-1, and PNP-2, were analyzed. Mechanical tests were performed by Universal Testing Machine (UTM; MCT-1150, AND Inc., Japan) at a constant speed of 10 mm/min. The hydrophilicity of nanofibers was evaluated by depositing deionized water droplets directly on the nanofiber surfaces, and the measurements were recorded using a contact angle instrument (Phoenix-MT).

### Degradation and swelling study

To analyze the degradation and swelling of PVA composite nanofibers, PVA, PNP-0.5, PNP-1, and PNP-2 nanofibers were each cut into 1 × 1 cm pieces and immersed in 1×PBS. The nanofibers, kept at room temperature, were removed at predetermined time intervals, then dried in an oven. The weight of the dried samples was recorded, and the degradation (%) was calculated using Eq. (1):

1 $$\:Degradation\left(\%\right)=\:\frac{{W}_{o}-{W}_{t}}{{W}_{o}}\:\times\:100$$

Where *W*_*o*_ is the weight of the dried nanofibers before experiment and *W*_*t*_ is the weight of the dried nanofibers after degradation.

At specific time intervals, the excess water was removed using filter paper and weighed, then immersed back into the 1×PBS. The swelling (%) was calculated using Eq. (2):

2 $$\:Swelling\left(\%\right)=\:\frac{{W}_{s}-{W}_{d}}{{W}_{d}}\:\times\:100$$

Where *W*_*s*_ is the weight of the swollen nanofibers and *W*_*d*_ is the weight of the dried nanofibers.

### In vitro photothermal reaction

The in vitro photothermal performance of PVA and PNP-0.5, PNP-1, and PNP-2 was investigated under exposure to 808 nm NIR laser light (MDL-H-808–5 W, BL23255) purchased from Changchun New Industries Optoelectronics Tech Co., Ltd, China. The photothermal effects were examined under 0.5 and 1.0 W/cm^2^ power densities. The real-time temperature was measured at 1 min intervals over a 5 min period. The images were obtained using a thermal camera (FLIR Ex-Series (E6-XT)).

### Detection of ROS

Hydroxyl radicals generated in ND and ND@PDA nanoparticles (0.1 mg/mL) upon NIR irradiation were detected using electron spin resonance (ESR) spectroscopy with a JEOL (JES-X320) spectrometer equipped with a xenon lamp. 2,2,6,6-Tetramethylpiperidine (TEMP) was employed as the spin-trapping agent. Each sample was analyzed before and after 5 min of NIR irradiation.

### Curcumin release test

The curcumin release test was performed for pure PVA/cur and PNPC nanofibers. For this experiment, 2 × 2 cm of PVA/Cur and PNPC nanofibers were incubated in 10 mL 1×PBS (pH 7.4) containing 2% Tween at 37 °C and 100 rpm. At predetermined time intervals (0.5, 2, 4, 6, 12, 24, 48, 96 h), medium was extracted, and an equal amount of fresh 1×PBS was added. To assess the effect of NIR on curcumin release, NIR was irradiated on the samples in 1×PBS solution before every extraction.

This experiment was repeated three times (*n* = 3) for accurate analysis. The collected medium was measured at a wavelength of 425 nm using a UV-vis spectrophotometer (Infinite^®^ M Nano 200 Pro; TECAN, Switzerland). The released curcumin was calculated using Eq. (3):

3 $$\:Drug\:release\left(\%\right)=\:\frac{{D}_{released}}{{D}_{total}}\times\:100$$

Where *D*_*released*_ is the total amount of curcumin released, and *D*_*total*_ is the total amount of curcumin incorporated in the nanofibers.

### In vitro antibacterial activity

In this experiment, Gram-positive Methicillin-resistant *Staphylococcus aureus* (MRSA) and Gram-negative *Escherichia coli* (*E. coli*) bacteria were utilized. All bacteria were tested on PVA, PNP-2, and PNPC samples before and after NIR treatment. MRSA and *E. coli* were activated by culturing overnight in sterile nutrient broth at 37 °C, 100 rpm, and were used for further experiment after adjusting OD_600_ = 0.1 (100 µL). Simultaneously, the 30 mg samples were sterilized under UV light for 2 h prior to the experiment. A 1 mL bacterial suspension was cultured with the samples to evaluate the antibacterial activity. The treatment group was irradiated with 1.0 W/cm^2^ NIR for 5 min before incubation for 6 h. While the control group was incubated under the same conditions without irradiation. After 6 h, the suspension diluted to 10^− 5^ was cultured on nutrient agar plates at 37 °C overnight, and the antibacterial efficiency was determined by following Eq. (4).

4 $$\begin{aligned}\:Antibacterial\:efficiency&(CFU/plate)\left(\%\right)\\ & \quad=\:\frac{{CFU}_{control}-{CFU}_{Treatment}}{{CFU}_{control}}\times\:100\end{aligned}$$

where *CFU*_*control*_ is the total CFU in the control group and *CFU*_*Treatment*_ is the total CFU in the experimental group.

To observe the morphology of bacteria in the control and treatment groups, the bacteria were washed with 1×PBS and then fixed with 4% paraformaldehyde for 1 h. Subsequently, the bacteria were dehydrated with a series of ethanol at concentrations of 30, 50, 70, 80, and 100%, respectively. The prepared samples were further dried overnight and gold-coated for SEM.

### Live/dead staining

The control set and NIR-irradiated bacteria samples were further analyzed by live/dead staining. The bacteria were incubated for 15 min with 1 µL each of SYTO9 dye (25 mM) and propidium iodide (1.5 mM). Live/dead images were then captured with a fluorescence microscope (DMi8 Series, Leica Microsystems, Germany).

### Anti-biofilm activity

To examine anti-biofilm activity, the same bacteria, *E. coli* and MRSA, were used. The biofilm was prepared in a 24-well plate by incubating 200 µL of *E. coli* and MRSA fresh cultures (OD_600_ = 0.1) with 1 mL of sterile nutrient broth and tryptic soy broth, respectively. The cultures were grown at 37 °C for over 2 days for robust biofilm formation. Next, the old media was removed and carefully replaced with fresh media containing PVA, PNP-2, and PNPC samples and were incubated for an additional 6 h. After 6-h incubation, the treatment group was exposed to NIR light at 1.0 W/cm^2^ for 5 min.

Quantitative analysis was performed using the crystal violet assay. The supernatant was removed, and the biofilm formed in the wells was gently washed with 1×PBS. Each well was then stained with 500 µL of 0.1% crystal violet at room temperature for 15 min. Excess stain was removed by carefully washing with 1×PBS, and the wells were air-dried. Images of the stained biofilm remaining on the well bottoms were captured. Subsequently, 500 µL of 33% acetic acid was added to solubilize the stain, and the absorbance of the solution was measured at 595 nm.

### In vitro assay

#### Cell culture

Human dermal fibroblasts (HDF) (ATCC No. PCS-201-012) cells were used for cell experiments. The growth medium consisted of DMEM supplemented with 10% FBS and 1% P/S antibiotics. HDF cells were cultured in DMEM at 37 °C with 5% CO_2_ until confluence. The cultural media was changed every 2–3 days. Before seeding onto the samples, the cells were counted using a hemocytometer.

#### Cytotoxicity assay of nanofibers

The cell viability of HDF was evaluated on PVA, PNP-2, and PNPC nanofibers containing media. Nanofibers were cut into 0.5 × 0.5 cm pieces, washed with 1×PBS, and sterilized by UV treatment before being placed in a 96-well plate. Each well was seeded with 0.5 × 10^4^ cells/well. Cell viability was assessed on days 1, 3, and 5 after seeding, and media was changed every 2 days. Prior to analysis, old media was discarded, and cells were washed with 1×PBS following the addition of 10% WST-8 dye in fresh media. The cells were further incubated for 2 h at 37 °C to form formazan. The WST-8 kit contains tetrazolium salt, which converts to orange formazan dye in the presence of viable cells. Therefore, a higher number of live cells produces more formazan dye. After 2 h, optical density (OD_460_) was measured using UV-vis spectrophotometer (Infinite^®^ M Nano 200 pro; TECAN, Switzerland). All experiments were performed in triplicate (*n* = 3) for accuracy.

#### Live/dead assay

UV-treated nanofibers were placed in a 48-well plate, and 1 × 10^4^ HDF cells/well were cultured on nanofibers and incubated at 37 °C. Every 2 days, the old medium was replaced with fresh medium. Before analysis, the cells were washed with 1×PBS and then treated with 1 µL of acridine orange and ethidium bromide dye in a 1:1 ratio per well. Live/dead images were captured on days 1, 3, and 5 using a fluorescence microscope (DMi8 Series, Leica Microsystems, Germany).

#### Cell morphology

Sterilized nanofibers cut into 1 × 1 cm pieces were placed in a 48-well plate, and HDF cells (0.5 × 10^4^ cells/well) were cultured on it. After culturing the cells for 5 days, they were fixed at room temperature with 4% paraformaldehyde for 15 min. Following fixation, the PFA was removed, and 0.1% TritonX-100 was added, incubating for 10 min. Subsequently, the cells were blocked with 1% BSA under shaking conditions for 1 h. Next, the samples were incubated with Actin green and rhodamine dye for 10 min to stain the actin filaments of cells. The cells were washed with 1×PBS until unwanted dye spots were removed. 4’, 6-diamidino-2-phenylindole dihydrochloride (DAPI) was used to stain the nuclei, incubating for 2 min. Mounting media was added to protect the cells. The morphology of the stained cells was imaged using a fluorescence microscope.

### Statistical analysis

The statistical analysis was conducted using the OriginPro 9.0 software. The statistical significance between the control and treatment groups was assessed using one-way analysis of variance (ANOVA). The data is displayed as mean ± standard deviation (SD). Significant differences were determined at a level of statistical significance of *p < 0.05, **p < 0.01, ***p < 0.001.

## Results and discussion

### Characterization of ND hybrids

**Fig. 1:**
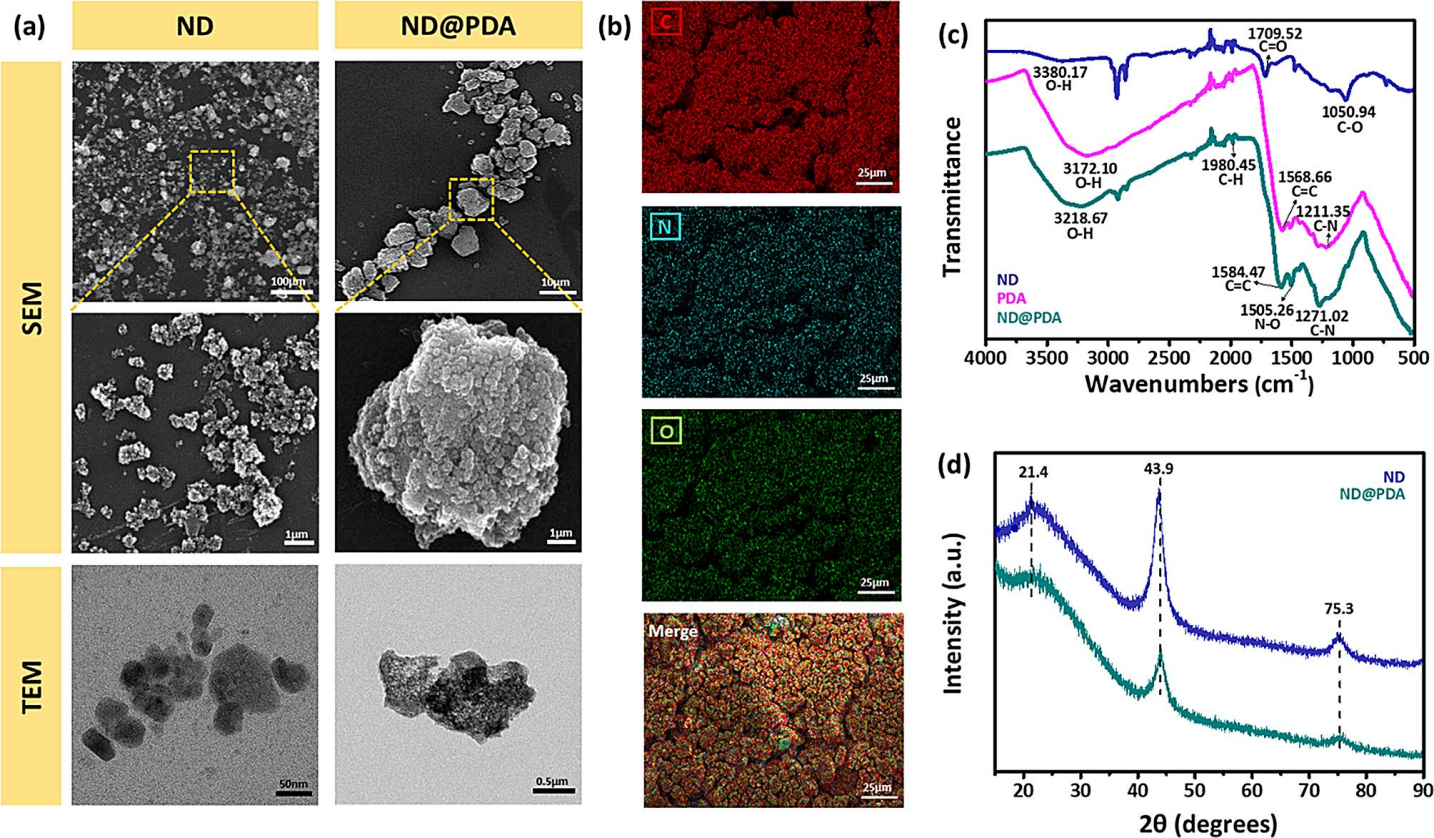
Nanoparticle characteristics: **(a)** SEM and TEM images of ND and ND@PDA **(b)** EDS of ND@PDA showing the composition of C, N, O, and Merge **(c)** FTIR spectra of ND, PDA, and ND@PDA **(d)** XRD patterns of ND and ND@PDA

The schematic representation of the synthesis of ND@PDA is depicted in Scheme 1. ND@PDA was successfully synthesized using DA and ammonia solution. The synthesis was confirmed using characterization techniques, including SEM, EDS, FTIR, and XRD. Figure 1(a) shows SEM images of ND and ND@PDA. ND particles exhibited a typical irregular and aggregated morphology, consistent with previous studies [22, 23]. In the SEM image of ND@PDA, a well-coated layer of PDA on ND particles was observed. Indicating a successful deposition of the PDA layer on the surface of ND. The elemental composition was attained by EDS mapping (Fig. 1(b)). It was confirmed that C, N, and O are homogeneously distributed on the surface of ND@PDA. This indicates the effective coating of PDA on the surface of ND. Figure 1(c) shows the FTIR spectrum of ND, PDA, and ND@PDA. In ND, the absorption peaks at 3380.17, 1709.52, and 1050.94 cm^-1^ correspond to the stretching vibrations of the O-H, C = O, and C-O groups (oxygen-containing functional groups), respectively. While in PDA, absorption peaks at 3172.10, 1980.45, 1568.66, and 1211.35 cm^-1^ correspond to the stretching vibrations of the O-H, C-H, C = C, and C-N groups, respectively. In ND@PDA, new absorption peaks at 1980.45 and 1505.26 cm^-1^ corresponding to the stretching vibrations of the C-H and N-O groups, appeared, which confirmed the formation of new bonds between ND and PDA. Additionally, the O-H stretching has distinctly shifted from 3380.17 to 3218.67 cm^-1^. This is a result of the robust van der Waals interaction and π-π interaction and between PDA and ND [24]. The FTIR analysis results provided information confirming the successful synthesis of ND and ND@PDA. The chemical interaction between ND and PDA is demonstrated in Figure S2. XRD analysis was conducted to characterize the crystalline structure of ND and ND@PDA particles (Fig. 1(d)). ND particles exhibited a sharp diffraction peak at approximately 2θ = 21.4°, indicating the presence of sp^2^ carbon. Additionally, peaks at around 2θ = 43.9° and 75.3° indicated the presence of sp^3^ carbon [25]. In ND@PDA, each peak noticeably weakened, indicating a reduction in the carbon content of both sp^2^ and sp^3^ due to the surface coating of PDA [26].

**Fig. 2:**
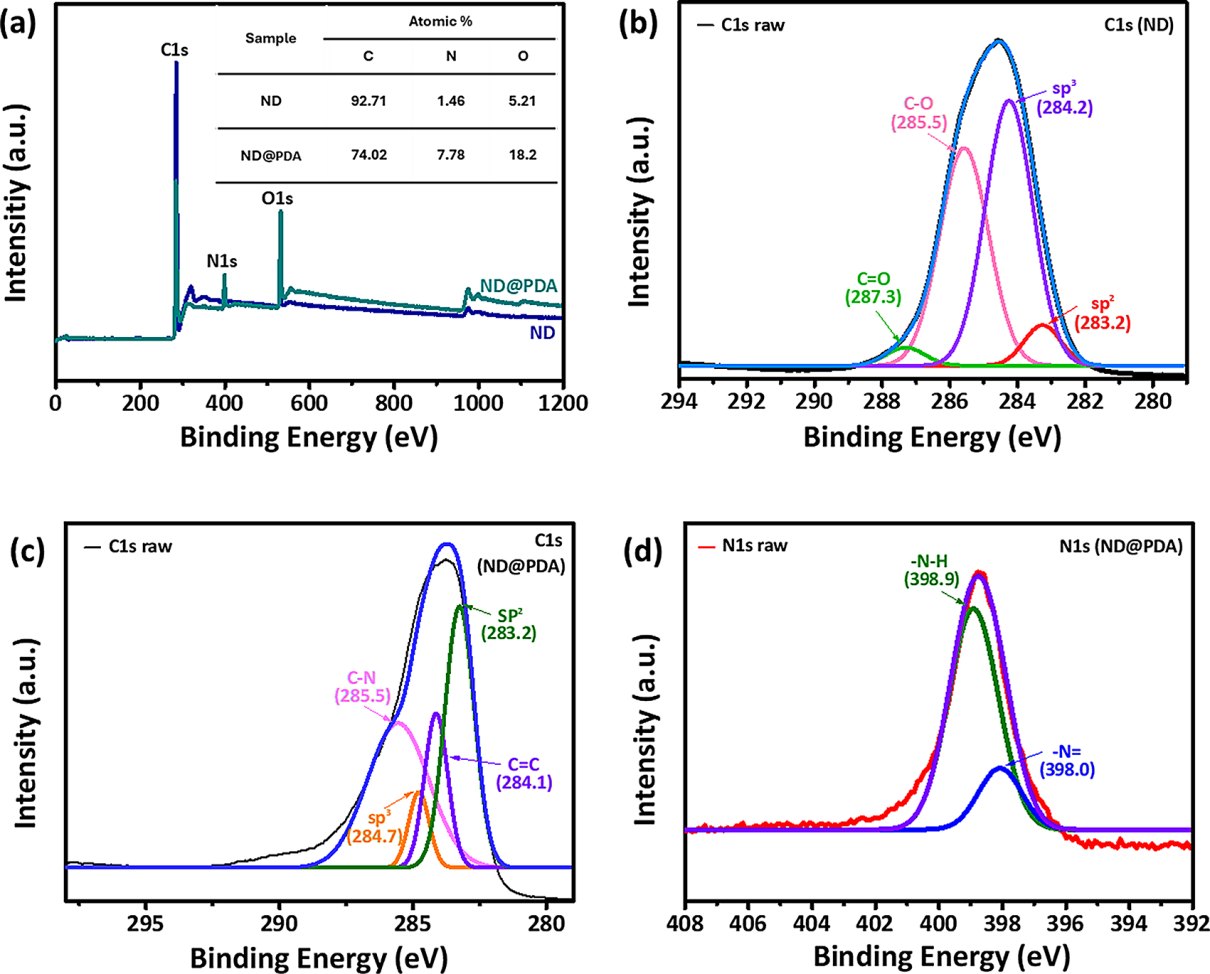
XPS spectra of ND and ND@PDA: **(a)** Wide scan spectrum of ND and ND@PDA **(b)** C1s core level spectrum of ND **(c)** C1s core level spectrum of ND@PDA **(d)** N1s core level spectrum of ND@PDA

The XPS spectra provided additional evidence to demonstrate the formation of a PDA layer on the ND surface. As shown in Fig. 2(a), sharp peaks of carbon and oxygen were detected in ND and ND@PDA. However, a weak N1s peak can be observed, which makes it clear the presence of nitrogen by polydopamine. Moreover, the atomic % of nitrogen atoms increased from 1.46 to 7.78%, and that of oxygen increased from 5.21 to 18.2%, indicating that PDA successfully coated ND particles. As evident in Fig. 2(b), the C1s core level spectrum of ND is deconvoluted into four peaks. The strongest peak is at 284.2 eV, originating from the diamond-like cluster associated with alkyl groups and sp^3^ carbon. Additionally, a peak is observed at 283.2 eV for the sp^2^ carbon. Furthermore, peaks corresponding to C-O and C = O groups at 285.5 and 287.3 eV, respectively, were found to be consistent with FTIR spectra. The N1s peak in ND@PDA is observed at 398.9 eV (Fig. 2(c)). In the N1s core level spectrum of ND@PDA, two distinct peaks were identified (Fig. 2(d)). The peaks are 398.0 and 398.9 eV, respectively, corresponding to the -N = C group formed during the reaction of dopamine molecules to form polydopamine and the N-H group of dopamine itself. All these results collectively indicate that PDA was successfully coated on ND particles.

### Characterization of nanofibers

**Fig. 3:**
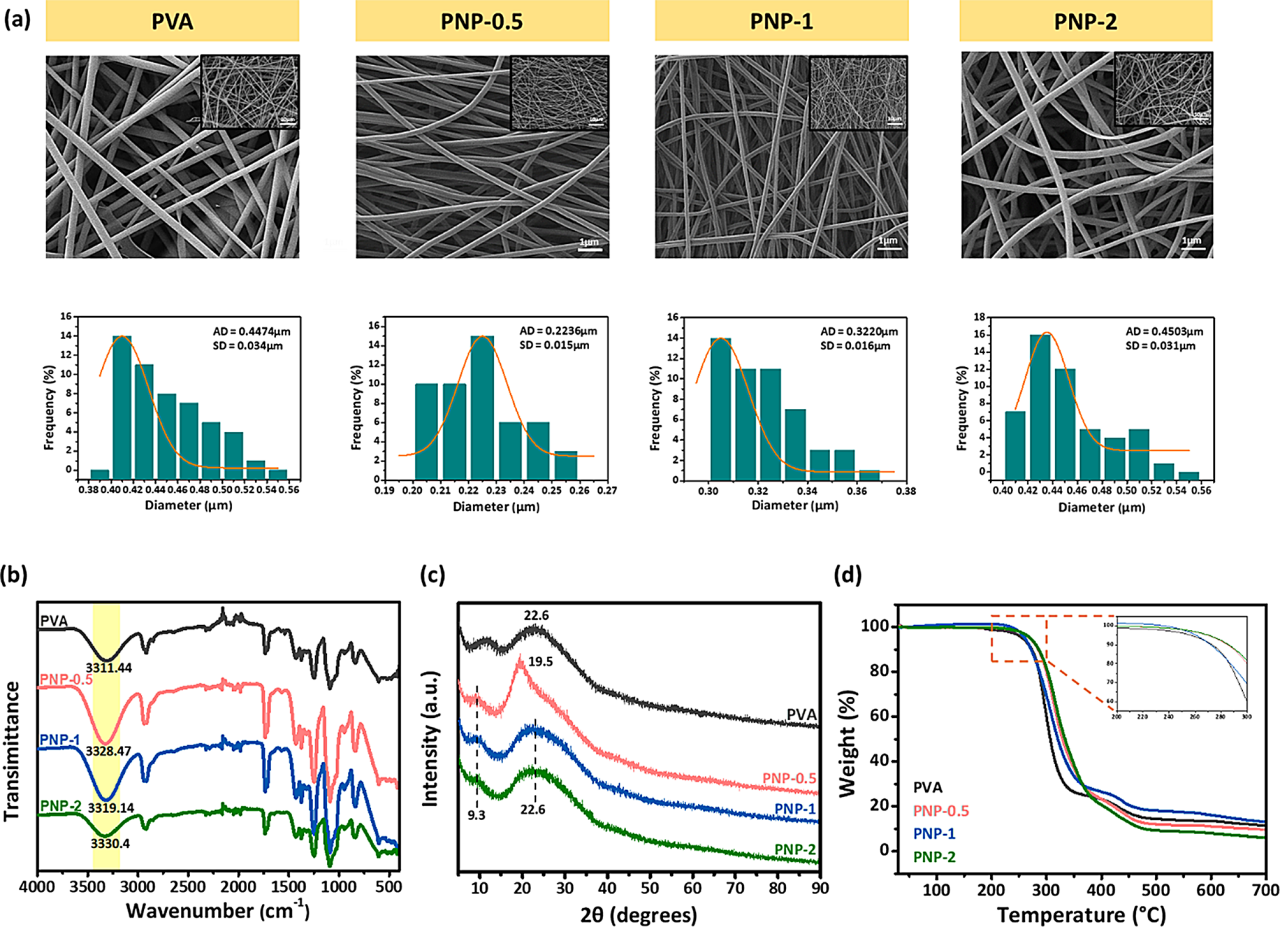
Physical and chemical properties of nanofibers: **(a)** SEM morphology of PVA and PNP nanofibers with their size distribution, **(b)** FTIR spectra, **(c)** XRD and **(d)** TGA

The surface morphology of PVA and PNP-0.5, PNP-1, and PNP-2 nanofibers was analyzed using SEM (Fig. 3(a)). The PVA nanofibers and PVA nanofibers containing ND@PDA exhibit similar shapes and smooth surfaces. The size distribution of the nanofibers was calculated using the ImageJ program. PVA nanofibers showed an average diameter of 0.45 ± 0.03 μm. The average diameters of PNP-0.5, PNP-1, and PNP-2 were 0.22 ± 0.02 μm, 0.32 ± 0.02 μm, and 0.45 ± 0.03 μm, respectively, showing an increasing trend with the concentration of ND@PDA. PNP-2 exhibited the most similar average diameter to pure PVA. PVA contains numerous hydroxyl (-OH) groups, enabling it to form intra- or intermolecular hydrogen bonds [27, 28]. FTIR was performed to investigate the relationship between PVA and ND@PDA, and the results are depicted in Fig. 3(b). The -OH peak of PVA was observed at 3311.44 cm^-1^. Additionally, the peaks for PNP-0.5, PNP-1, and PNP-2 were found at 3328.47, 3319.14, and 3330.45 cm^-1^, respectively. This indicates the presence of hydrogen bonding between the hydroxyl groups of PVA and the catechol groups present in PDA. The XRD patterns of PVA and nanofibers containing ND@PDA from 5° to 90° are shown in Fig. 3(c). Pure PVA nanofibers exhibit a typical diffraction peak at 2θ = 22.6°, indicating a semicrystalline structure with both crystalline and amorphous phases [29, 30]. The same peak is observed in PNP-1 and PNP-2, suggesting that the addition of ND@PDA does not significantly impact the polymer’s crystalline structure. However, PNP-0.5 shows a distinct sharp diffraction peak at 2θ = 19.5°, indicating that with a lower concentration of ND@PDA, the crystalline structure of the nanofibers changes. Additionally, a 2θ = 9.3° peak attributed to the amorphous nature of polydopamine was observed in the PNP nanofibers, confirming the presence of ND@PDA. This indicates that the PDA coating on the ND surface was successfully achieved, and this characteristic is also reflected in the PNP nanofibers.

### TGA

The analysis of the thermal stability of pure PVA and PNP-0.5, PNP-1, and PNP-2 nanofibers is presented in Fig. 3(d). The maximum thermal degradation stage was observed at 200–300 °C, which is consistent with previous reports [31]. Compared to pure PVA nanofibers with a maximum decomposition temperature of 229.24 °C, the PNP-2 nanofibers exhibited a small increase in decomposition temperature (233.13 °C). This suggests no significant change in the thermal stability and demonstrate steady structure of PNP nanofibers.

### Mechanical testing

**Fig. 4:**
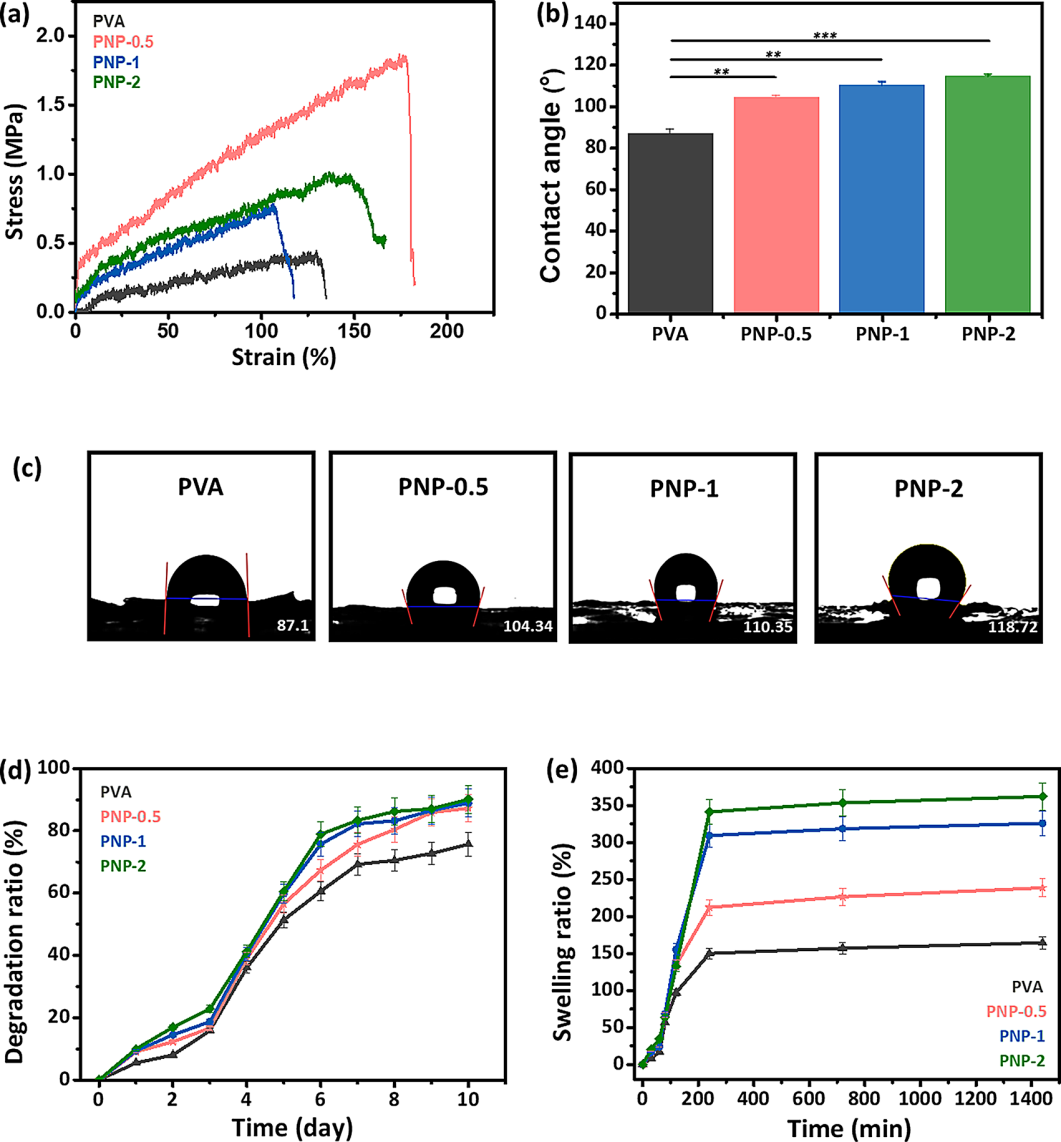
Mechanical properties of PVA and PNP nanofibers: **(a)** Stress-strain curves **(b**,** c)** Contact angle **(d)** Degradation study and **(e)** Swelling

Mechanical testing was performed on the PVA, PNP-0.5, PNP-1, and PNP-2 samples. Figure 4(a) shows the stress-strain curves for each of the samples. Pure PVA exhibited a very low elongation at break, but the addition of ND@PDA increased the elongation at break. This enhancement in ductility is due to strong bonding formed between ND@PDA and the nanofibers. Such interactions reinforce the matrix structure of the nanofibers and result in a more uniform stress distribution, leading to an increased elongation at break [32].

### Contact angle

The contact angle of PNP nanofibers was measured to evaluate the impact of ND@PDA on the wettability of PVA. PVA is widely used in various applications, especially in medical and pharmaceutical fields, due to its excellent properties [33]. The contact angle of pure PVA was confirmed to be 87.1°, indicating its hydrophilic surface properties. As seen in Fig. 4(b) and (c), PNP-0.5, PNP-1, and PNP-2 exhibited contact angles of 104.34°, 110.35°, and 118.72°, respectively. These values indicate that the samples showed hydrophobic characteristics. The results suggest that the addition of ND@PDA significantly affects the wettability of the nanofibers. A trend of increasing contact angle was observed with higher concentrations of ND@PDA.

### Degradation and swelling profile

The degradation and swelling profiles of PVA composite nanofibers are shown in Fig. 4(d, e). The degradation rate of PNP nanofibers was higher than that of pure PVA (Fig. 4(d)). After 10 days, the degradation rates of PNP-0.5, PNP-1, and PNP-2 nanofibers were 87.2%, 88.9%, and 90.1%, respectively, showing higher rates with increased concentrations of ND@PDA. In terms of swelling rate, after 4 h, PNP-0.5, PNP-1, and PNP-2 exhibited swelling rates of 212.2%, 309.2%, and 341.4%, respectively, compared to 150% for PVA nanofibers (Fig. 4(e)). These results indicate that the addition of ND@PDA to the nanofibers enhances the degradation and swelling potential of the composite material. Consequently, the increased degradation and swelling rates compared to PVA nanofibers can promote wound healing and prevent infections, making them suitable for application in wound healing platforms [34–36].

Based on the findings so far, we have determined that PNP-2 is suitable for our experimental purposes of antibacterial performance and wound healing. Therefore, we further considered adopting PNP-2 for future experiments.

### Photothermal reaction

**Fig. 5:**
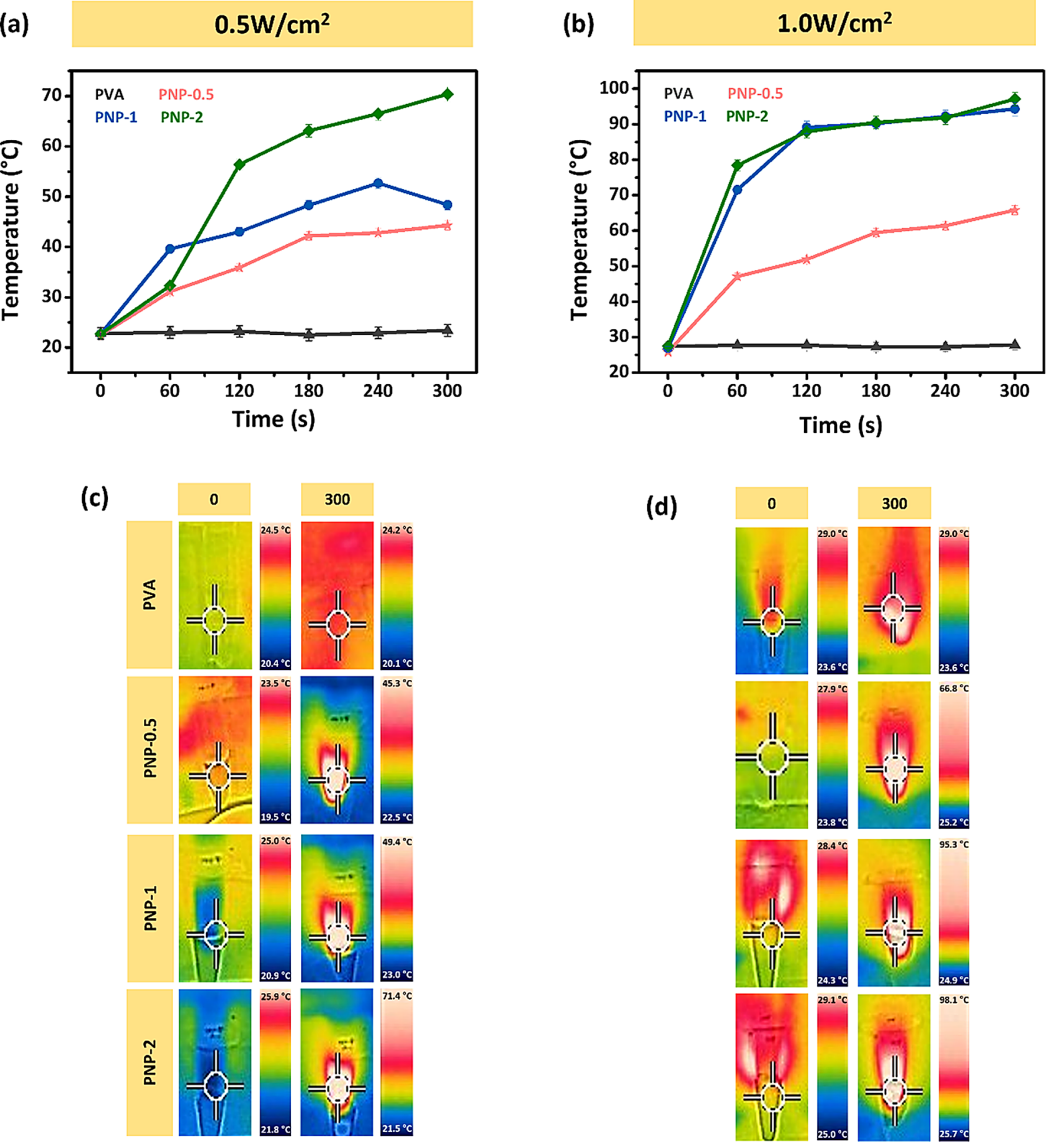
Photothermal properties: Temperature change in nanofibers at **(a)** 0.5 W/cm^2^ and **(b)** 1.0 W/cm^2^**(c**,** d)** Infrared thermal images on irradiation of 0.5 W/cm^2^ and 1.0 W/cm^2^

NIR light, with a wavelength range of 750 to 2500 nm, has a longer wavelength than visible light and is often referred to as the biological window. Due to its high penetrability, NIR light can penetrate biological tissues to a maximum depth, offering significant advantages such as minimal damage and enhanced therapeutic effects. Additionally, NIR light irradiation using photothermal materials can effectively kill bacteria through the generation of ROS [37–39]. To evaluate the photothermal response of PVA and PNP, samples were irradiated with 808 nm NIR light. The samples were assessed at 0.5 and 1.0 W/cm^2^ power density. Figure 5(a) and (c) show the temperature increase of the samples under 0.5 W/cm^2^ NIR light, while Fig. 5(b) and (d) display the temperature increase under 1.0 W/cm^2^ NIR light after 5 min of irradiation. The control sample, PVA, did not have any change in temperature under both 0.5 and 1.0 W/cm^2^ power density NIR light. PNP-0.5 showed a temperature rise compared to the control. However, the temperature increased moderately, from 22.5 °C to 44.3 °C at 0.5 W/cm^2^ and from 25.8 °C to 65.8 °C at 1.0 W/cm^2^. PNP-1 demonstrated a similar temperature rise curve to PNP-0.5 at 0.5 W/cm^2^, increasing from 22.7 °C to 48.4 °C, but at 1.0 W/cm^2^, the temperature rose significantly, from 26.9 °C to 94.3 °C. In contrast, PNP-2 exhibited the most rapid and substantial temperature increase, reaching 70.4 °C and 97.1 °C under 0.5 and 1.0 W/cm^2^ NIR light, respectively. This indicates that the ND@PDA-based samples have excellent photothermal performance. Based on these results, PNP-2 was ultimately selected for further use.

**Fig. 6:**
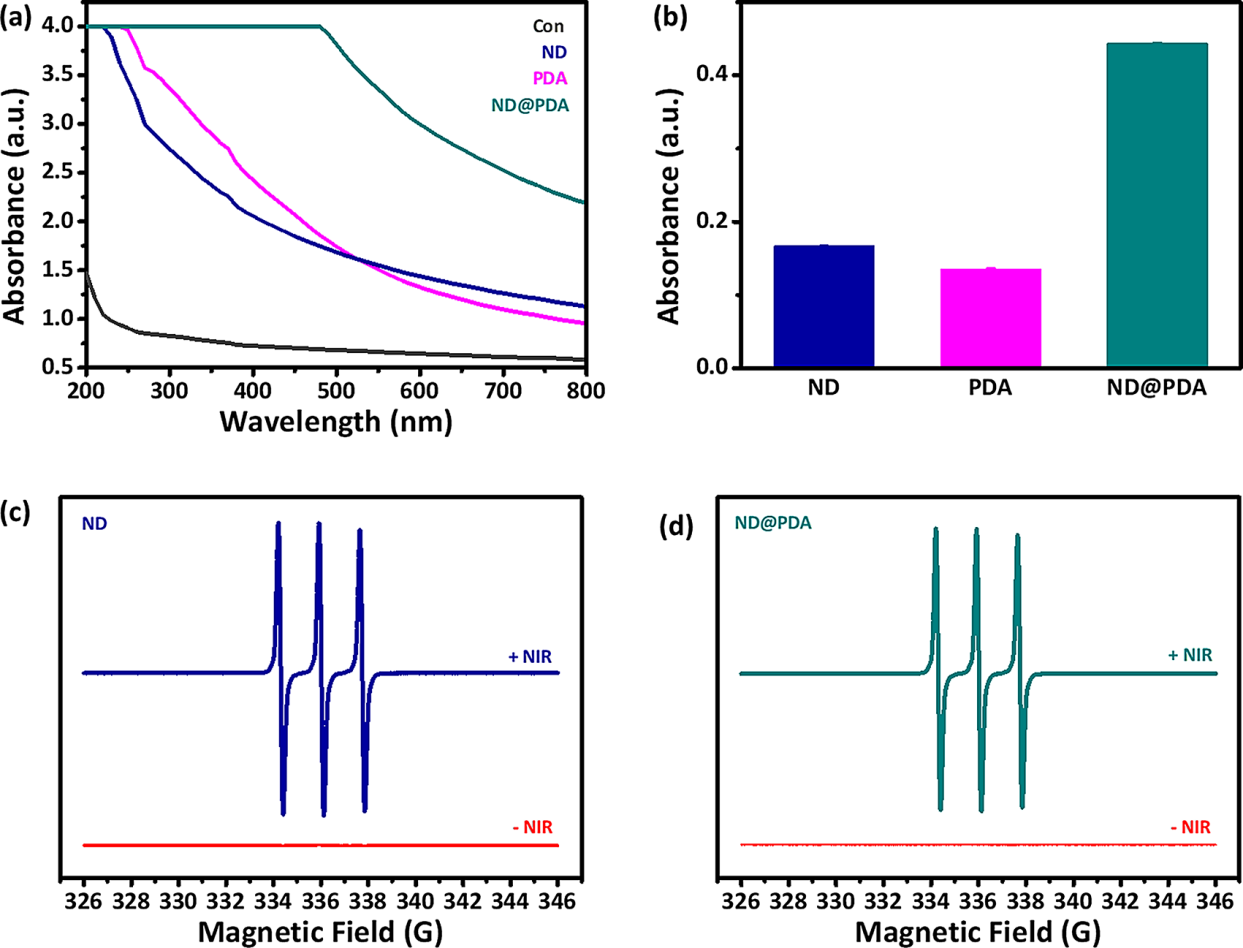
**(a)** UV-vis spectra of Control, ND, PDA, and ND@PDA **(b)** Absorbance of ND, PDA, and ND@PDA at 808 nm **(c**,** d)** ESR spectra of ND and ND@PDA with and without 1.0 W/cm^2^ NIR irradiation

The optical absorption capabilities of ND, PDA, and ND@PDA were carefully investigated using UV-vis spectroscopy. As shown in Fig. 6(a), control (DI) exhibited a very low absorbance value. In contrast, ND and PDA demonstrated stronger absorption capabilities in a wide range of wavelengths. PDA showed absorbance over a broader wavelength range compared to ND. This indicates that the broad absorption spectrum of PDA, combined with the properties of ND, results in a synergistic effect that enhances the absorption characteristics of ND@PDA. Figure 6(b) shows the absorbance of samples at 808 nm wavelength.

### Radical generation

Electron spin resonance (ESR) was employed to detect the generation of free radicals in ND and ND@PDA nanoparticles with and without NIR irradiation. As shown in Fig. 6(c), the NIR-treated ND samples exhibited strong triple peaks, which are characteristic peaks of singlet oxygen detected by TEMP trapping agents [40, 41]. Strong signals at g-factor values of 2.016, 2.006 and 2.005 were observed for ND. Similarly, in the case of ND@PDA, the NIR-treated samples showed ESR signals at g-factor values of 2.005, 2.016, and 2.015 as shown in Fig. 6(d). These results clearly indicate that the treated samples generated a high concentration of free radicals. However, no ESR signals were detected in ND and ND@PDA samples without NIR treatment, indicating that no free radicals were generated in the absence of NIR irradiation.

### Release of curcumin

**Fig. 7:**
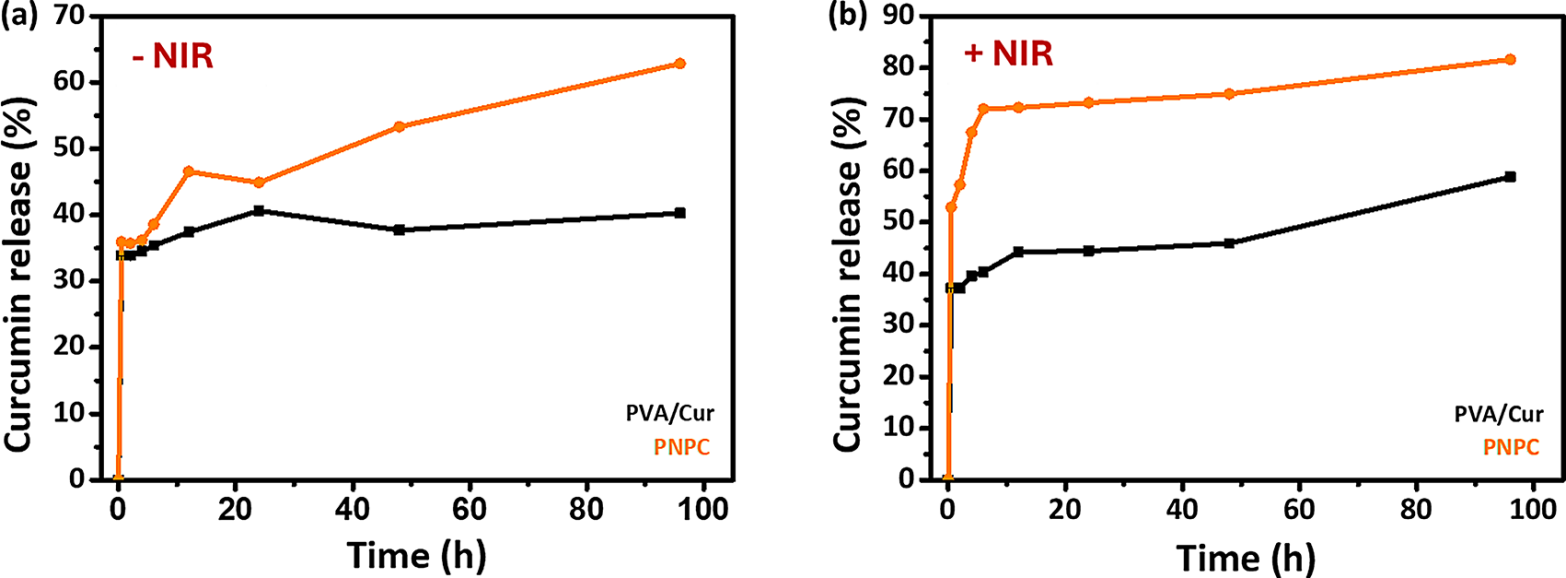
Curcumin release profile: **(a)** Without NIR and **(b)** With NIR

Curcumin is a naturally occurring polyphenol that is present in the rhizomes of Curcuma longa. It is well-known for its antibacterial, anti-inflammatory, and antioxidant activities. It accelerates wound healing by improving the rate of wound contraction. Because of these characteristics, curcumin release plays an effective role in wound healing mechanisms [42, 43]. To test curcumin release under NIR irradiation, 1% curcumin was incorporated into PVA and PNP-2 solution. The curcumin release profiles of PVA/Cur and PNPC were evaluated in 1×PBS with pH 7.4. As shown in Fig. 7(a), when NIR was not applied, both PVA/Cur and PNPC nanofibers exhibited only slight release, with no significant difference between them during the first 6 h. PVA/Cur demonstrated a tendency for gradual release over 24 h. After 48 h, there was a marginal decrease to 37.7%, but it subsequently reverted to the original release trend. PNPC nanofibers, however, displayed a rapid release from 38.6 to 46.5% after 12 h. The release slightly decreased to 44.9% in 24 h but continued at 53.3% and 62.9% at 48 and 96 h, respectively. Under NIR irradiation, PVA/Cur exhibited a release of 37.3% in 30 min, which was not significantly different from the rate before irradiation (Fig. 7(b)). It then showed a sustained release, reaching a higher release rate of 58.8% within 96 h compared to without irradiation. The PNPC nanofibers, on the other hand, released 53% of the curcumin quickly and significantly within 30 min, which was significantly higher than when they were not exposed to NIR radiation. The release continued continuously for 96 h. At 96 h, the release rate reached a notable 81.6%. The photothermal conversion ability of the ND@PDA nanoparticles under irradiation causes a rise in nanofiber temperature, which in turn causes an increase in curcumin release. NIR irradiation triggers the initial burst release of the drug, which aids in achieving the minimum effective concentration required for a rapid pharmacological response. This subsequently facilitates sustained drug release, thereby maximizing the therapeutic effect [44–46].

### Antibacterial performance

**Fig. 8:**
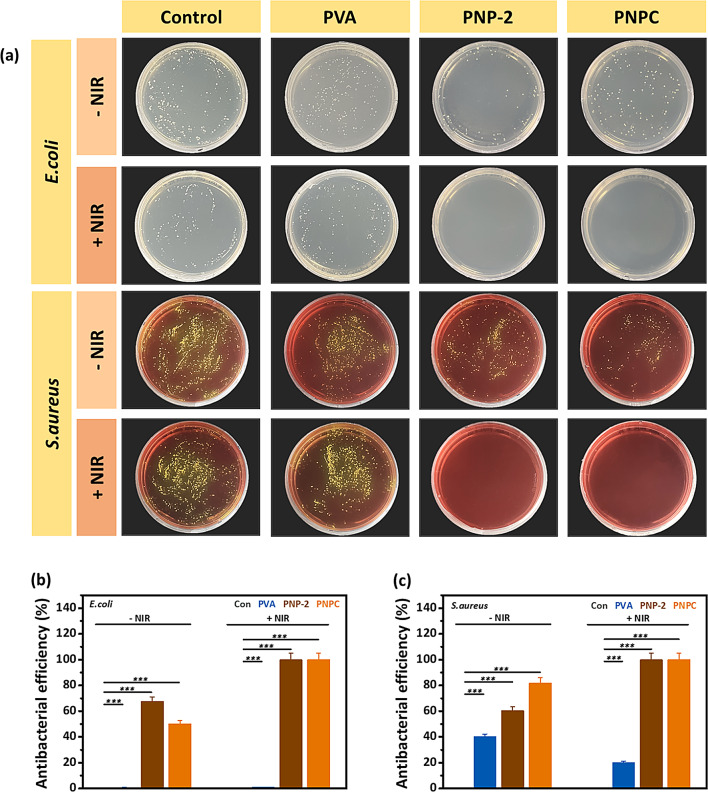
NIR-induced antibacterial effects of fabricated nanofibers: **(a)** Images of *Escherichia coli* and Methicillin-resistant *Staphylococcus aureus* bacterial colonies incubated for 6 h and their **(b**,** c)** Antibacterial efficiency percentage. Data are statistically significant at mean ± SD, not significant - ns, *p < 0.05, **p < 0.01, ***p < 0.001 in the triplicate experiment

Bacteria such as *E. coli* and *Staphylococcus aureus* can cause infections during the wound healing process. Unlike acute wounds, infected wounds generally require a prolonged healing period and may, in severe cases, not heal completely [47, 48]. Therefore, antimicrobial effects play a crucial role in preventing infections and enhancing the rate of wound healing [49, 50]. To evaluate the antibacterial effects of nanofibers under NIR irradiation, experiments were conducted against *E. coli* (Gram-negative) and MRSA (Gram-positive). The antibacterial rate was assessed by counting the number of bacteria colonies using the agar plating method. Figure 8(a) and (b) show the colony results on agar plates and the corresponding antibacterial efficiency graphs, respectively. For PVA nanofibers, there was no difference in antibacterial efficiency against *E. coli* regardless of NIR irradiation. However, in the case of PNP-2, when exposed to NIR, both bacteria exhibited 100% antibacterial efficiency, demonstrating a dramatic antibacterial effect. Similarly, PNPC nanofibers also showed excellent antibacterial effects after NIR treatment, with antibacterial efficiency increasing to 100% for both bacteria. However, 1% curcumin alone did not exhibit antibacterial effects (Figure S3). This indicates that 1% curcumin itself does not possess the desired antibacterial properties. Therefore, the superior antibacterial performance of PNP-2 and PNPC is attributed to the photothermal and ROS-generating antibacterial properties of ND@PDA nanoparticles.

**Fig. 9:**
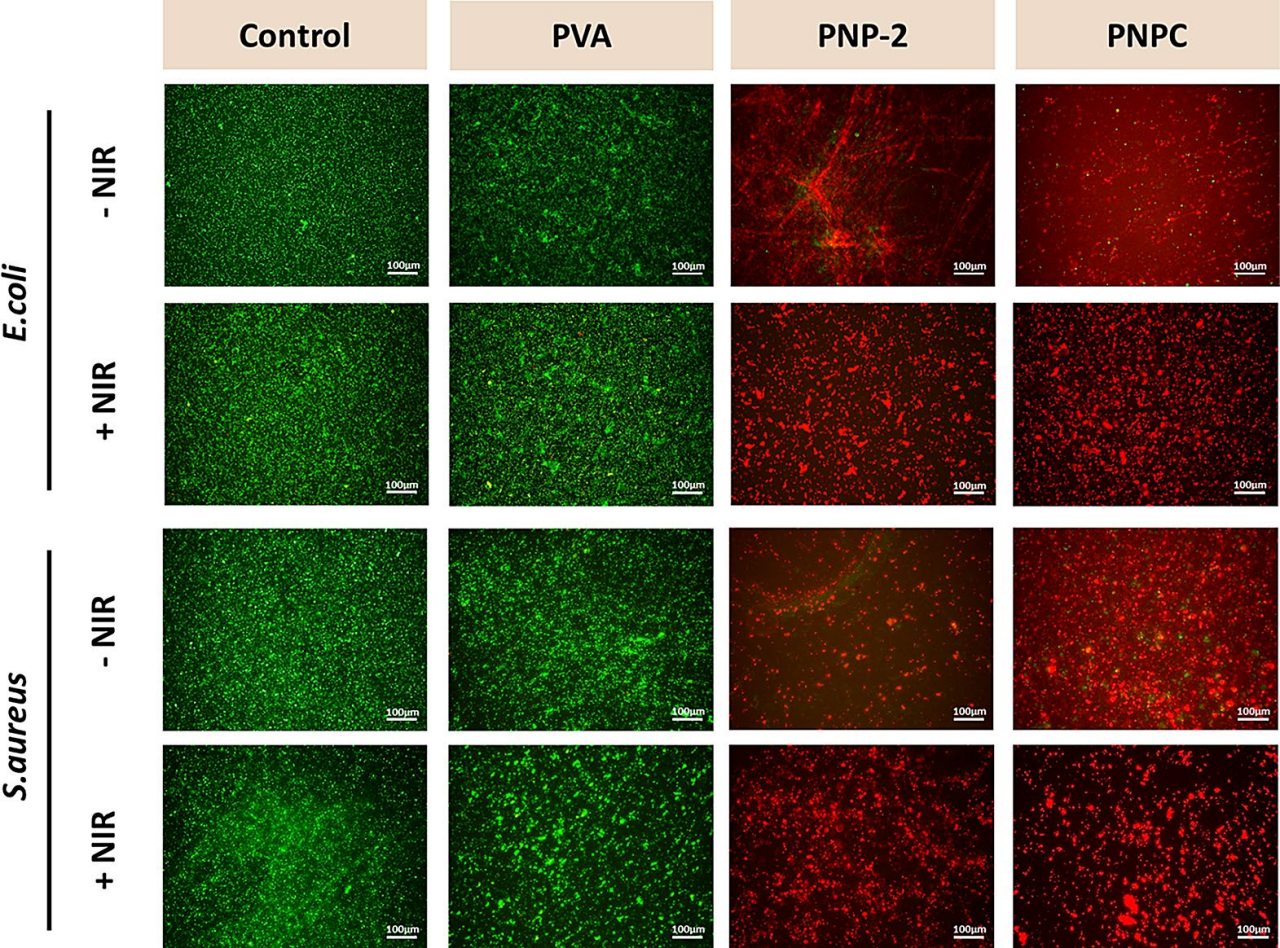
Fluorescent images of Live/dead of *Escherichia coli* and Methicillin-resistant *Staphylococcus aureus* cultured with nanofibers with and without irradiation of 1.0 W/cm^2^ NIR

Live/dead cell analysis using SYTO9/PI staining was employed to assess and confirm the membrane integrity of *E. coli* and MRSA (Fig. 9). Typically, SYTO9 binds to the DNA and RNA of all bacteria, regardless of membrane integrity, emitting green fluorescence. In contrast, PI enters only bacteria with damaged membranes, binding to their DNA and RNA and emitting a red fluorescent signal after merging both channels [51, 52]. The control and PVA still showed a substantial number of live bacteria even after NIR exposure, indicating the presence of healthy live bacteria. In contrast, the PNP-2 and PNPC nanofibers showed limited live bacteria before exposure, but a substantial number of dead bacteria were present. The low green signal observed in PNP-2 and PNPC is due to these two nanofibers significantly disrupting the membrane integrity of bacteria. PNP-2 and PNPC induce membrane damage, which limits the penetration of SYTO9, resulting in a reduced green signal. In contrast, the very high amount of red signals in PNP-2 and PNPC indicates a higher proportion of bacteria with damaged membranes, suggesting that a substantial amount of PI has bound to them. The quantitative analysis of live and dead bacteria in Figure S4 and S5 supports these findings. These results suggest that PNP-2 and PNPC effectively disrupt the membrane integrity of bacteria. The NIR exposure effectively killed the bacteria, demonstrating the presence of dead bacteria. These results suggest that nanofibers with ND@PDA effectively disrupt bacterial membrane integrity and enhance antibacterial efficacy.

**Fig. 10:**
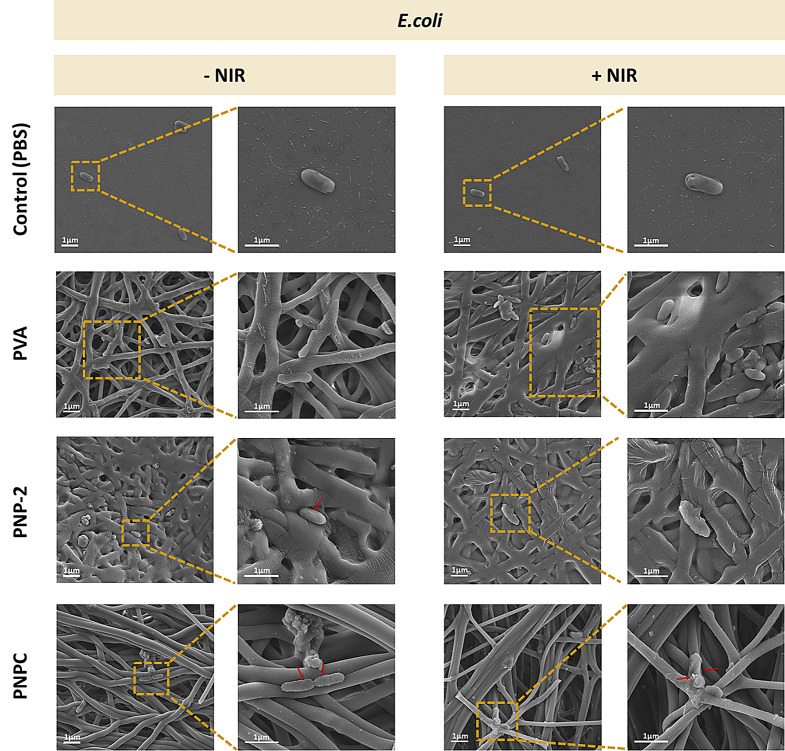
SEM images of *Escherichia coli* with and without irradiation of 1.0 W/cm^2^ NIR on nanofibers

To visually confirm the bacterial morphology, SEM analysis was performed on all samples before and after NIR treatment. Figure 10 shows that *E. coli* bacteria in the control group and on PVA nanofibers were rod-shaped and smooth before they were treated with NIR. However, after treatment, bacteria developed wall contraction and aggregated, losing their shape. In contrast, significantly fewer *E. coli* were observed on PNP-2 and PNPC nanofibers compared to PVA nanofibers, likely due to the PDA coating reducing bacterial adhesion [53]. Furthermore, we observed considerable wall contraction even before NIR treatment, resulting in rougher and more wrinkled forms compared to the control and PVA samples. After treatment, the bacterial membranes became even rougher and more ruptured.

**Fig. 11:**
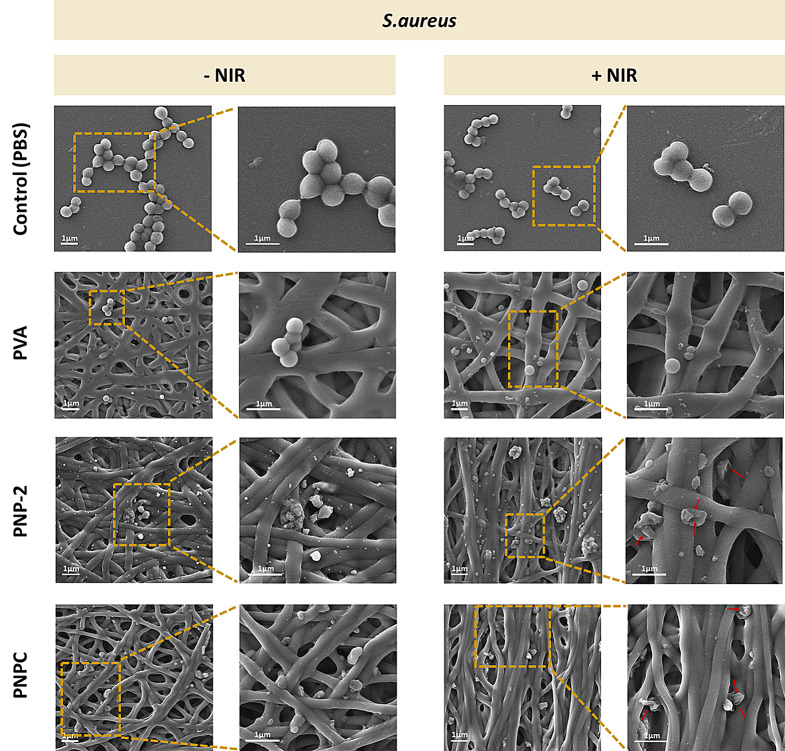
SEM images of Methicillin-resistant *Staphylococcus aureus* with and without irradiation of 1.0 W/cm^2^ NIR on nanofibers

For MRSA, bacteria in the control group maintained a smooth spherical shape very well without or with NIR treatment, while PVA showed only slight contraction (Fig. 11). Even before treatment, we observed significant contraction and shape deformation on PNP-2 and PNPC, likely due to PDA’s inherent antibacterial effect against various microorganisms [54]. After treatment, the degree of deformation increased, with some bacteria leaking their intracellular contents. In some cases, the bacterial morphology became unrecognizable. The higher deformation rate of MRSA compared to *E. coli* may be due to the effect of catechol groups, the photothermal effect, and oxidative stress. Gram-positive bacteria have a thick peptidoglycan layer, allowing catechol compounds to penetrate easily, whereas Gram-negative bacteria have a thicker outer membrane in addition to the peptidoglycan layer, making it more difficult for compounds to invade and interface with the antibacterial mechanism [55].

### Anti-biofilm activity

**Fig. 12:**
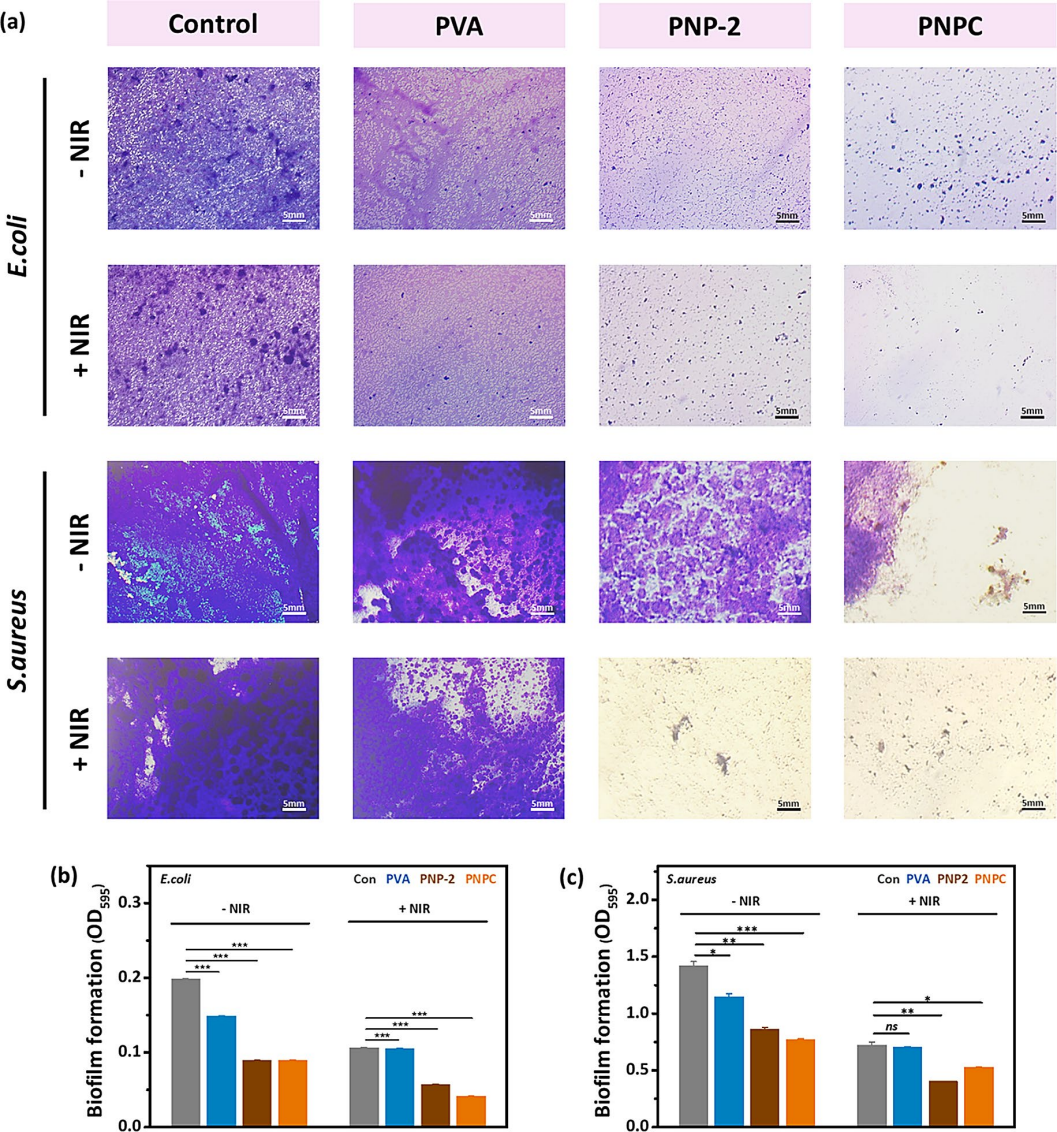
NIR-responsive antibiofilm property of nanofibers: **(a)** Biofilm degradation images with and without 1.0 W/cm^2^ NIR and their **(b**,** c)** Respective OD values. Data are statistically significant at mean ± SD, not significant - ns, *p < 0.05, **p < 0.01, ***p < 0.001 in the triplicate experiment

The formation of biofilms is a major cause of antibiotic resistance and can significantly impact chronic infections, leading to delayed wound healing [47, 48, 56]. To evaluate the efficacy of ND@PDA against biofilm formation, we investigated the biofilm degradation properties of nanofibers with and without NIR treatment. Figure 12(a) shows the crystal violet stain retained by biofilm with and without the treatment of NIR. Figure 12(b) and (c) demonstrate the absorbance value of *E. coli* and *S. aureus*, respectively. The crystal violet staining of NIR-treated and non-treated biofilm pictures did not show any change in the control and PVA nanofibers. However, PNP-2 and PNPC nanofibers exhibited minimal biofilm before NIR treatment, and the effect was highly noticeable after treatment. Only a very small amount of staining was observed on the wells, which shows that NIR treatment of PNP-2 and PNPC nanofibers can effectively eradicate biofilm formation. These results were possible because of the NIR absorption and photothermal potential of ND@PDA particles. This remarkable effect can, in turn, harm the biofilm’s EPS. The heat-induced damage leads to the collapse of the biofilm’s structural integrity and the dysfunction of EPS components, resulting in the removal of the biofilm [57, 58].

MRSA tends to produce robust and larger quantities of biofilm compared to other bacteria, making it more challenging to remove [59]. MRSA biofilms were also larger and denser than those of *E. coli*. Similar to *E. coli* outcomes, MRSA biofilms also showed no significant changes after NIR treatment in the control and PVA groups. However, PNP-2 and PNPC showed a significantly reduced biofilm after treatment compared to before. These results suggest that NIR treatment of PNP-2 and PNPC nanofibers has a significant impact on biofilm removal. This, in turn, could enhance antibiotic penetration and facilitate faster and more effective wound recovery. The biofilm results are consistent with the antibacterial results.

### Anti-biofilm performance mechanism of PNP nanofibers

**Scheme 2:**
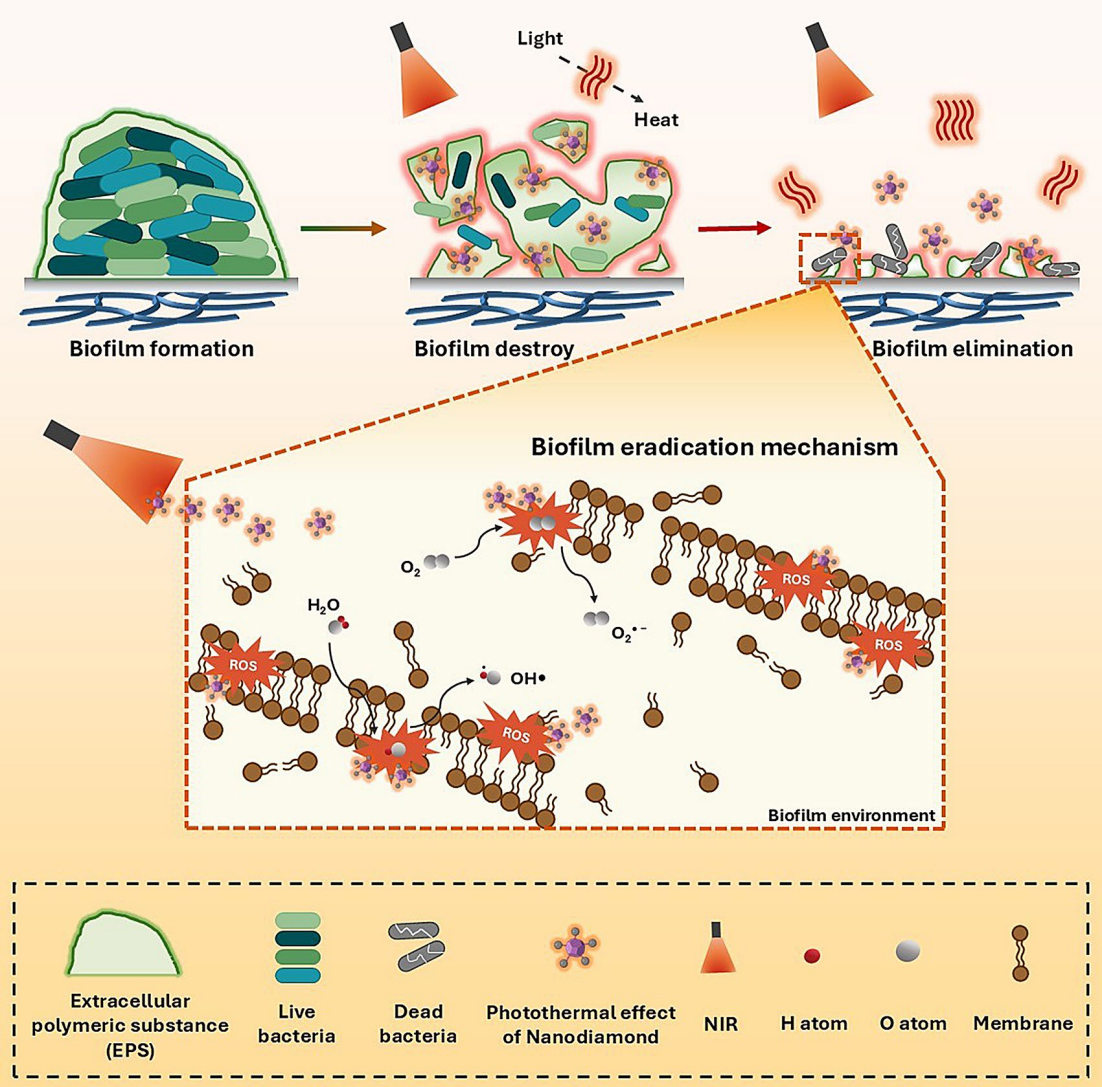
Schematic representation of the biofilm elimination and bacterial killing process of samples under NIR investigation

The ND@PDA nanoparticles embedded within PNP nanofibers possess photothermal properties that are activated upon exposure to NIR light. These ND@PDA nanoparticles absorb the NIR light and generate high levels of heat. This heat effectively disrupts the structural integrity of the biofilm by breaking down its key components, such as EPS, proteins, and lipids. The ND@PDA entering the destroyed biofilm environment also generates ROS (Scheme 2). The ROS produced by the NIR irradiation are highly reactive, oxidizing the proteins and lipids within the trapped bacteria, disrupting their metabolic activities, and leading to bacterial death. Moreover, ROS can cause DNA damage in bacteria, inhibiting replication and transcription, thus preventing bacterial proliferation. Continuous NIR irradiation ultimately results in the elimination of the biofilm and the eradication of the bacteria within. As a result, using PNP nanofibers along with NIR radiation is very effective at killing bacteria and biofilm.

### Cytotoxicity and live/dead assay

**Fig. 13:**
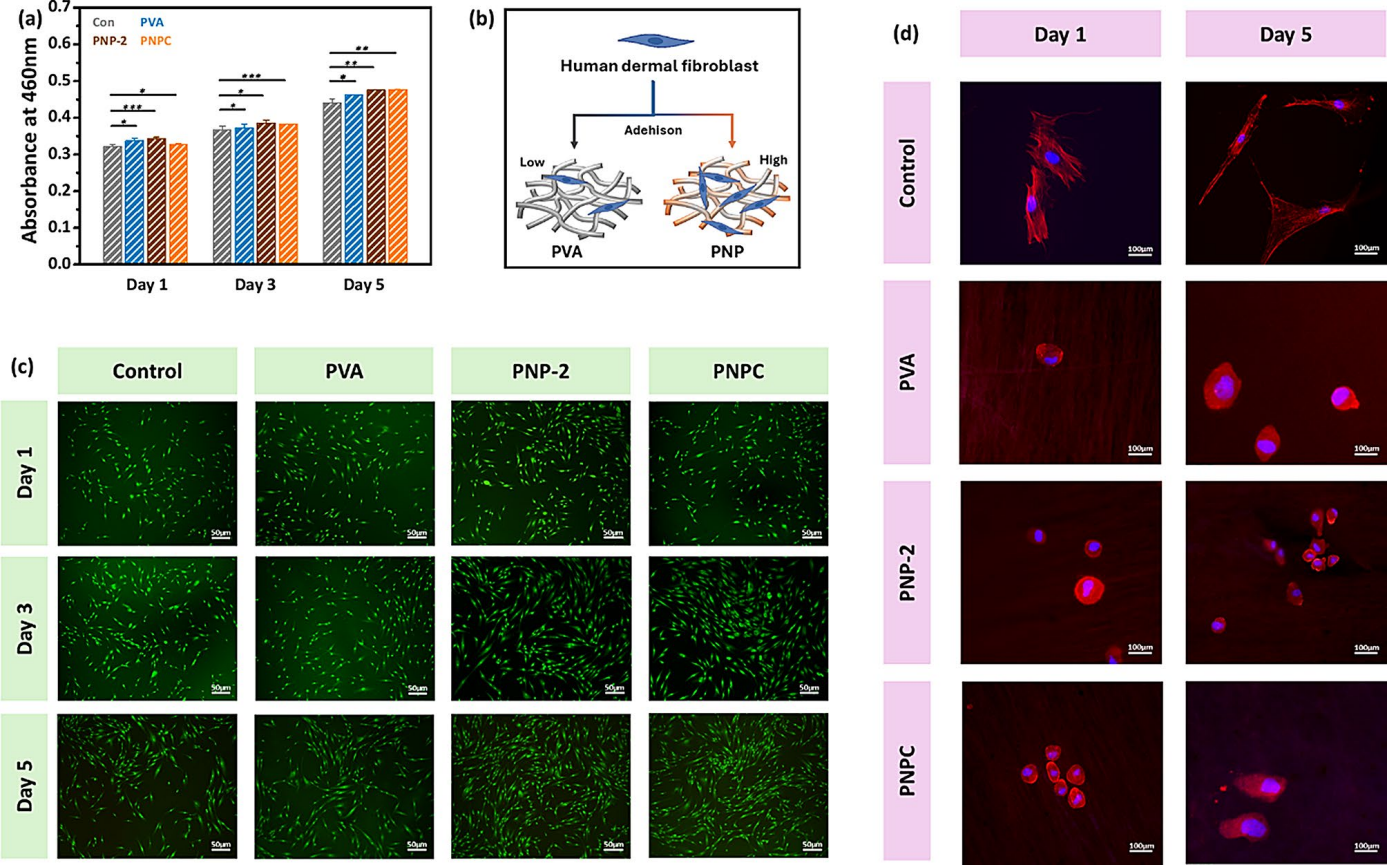
**(a)** Cell viability of HDF in presence of nanofibers evaluated from WST-8 assay for day 1, 3, and 5 **(b)** Schematic representation of cell adhesion on the nanofibers (red: actin cytoskeleton of cell; blue: DAPI stained nucleus) **(c)** Live/dead images of nanofibers for day 1, 3, and 5 **(d)** HDF morphology on nanofibers after 1 and 5 days. Data are statistically significant at mean ± SD, not significant - ns, *p < 0.05, **p < 0.01, *** *p* < 0.001 in the triplicate experiment

Excellent cell compatibility is a crucial factor in determining the suitability of wound healing scaffolds. The viability of HDF cells on the PNP nanofibers was assessed using the WST-8 assay. To determine if the nanofibers had any harmful effects on the cells, the cells were directly plated onto the nanofibers for 1, 3, and 5 days. The control group consisted of HDF cells plated without nanofibers. As shown in Fig. 13(a), all nanofibers exhibited noticeably improved cell growth rate compared to the control group except the PVA group. PNP-2 shows the fastest growth rate compared to PVA and control. A similar trend was observed on days 3 and 5. On day 5, the nanofibers containing ND@PDA continued to demonstrate higher cell viability than the control group. The findings suggest that the nanofibers containing ND@PDA are very compatible with HDF cells and help to stay alive.

Figure 13(c) shows the live/dead assay results for HDF cells on PNP nanofibers at days 1, 3, and 5. Overall, no cell death was observed across all samples, and notably, the PNP-2 samples exhibited significantly higher cell proliferation compared to the control and PVA samples. In the case of the PNPC samples, cell viability was similar to the control on day 1, but after day 3, cell proliferation increased significantly, much like the PNP-2 samples, when compared to the control and PVA. These results demonstrate that both PNP-2 and PNPC samples are non-cytotoxic, allowing cells to adhere well to the nanofiber surface and grow stably along its structure. This effect is likely due to the excellent cell adhesion and biocompatibility provided by the catechol (PDA)-functionalized ND, which enables cells to function normally [60, 61]. Additionally, PNPC showed no cytotoxicity, enabling cells to adhere well to the nanofiber surface and grow stably along its structure. A schematic of the cell adhesion properties of the nanofibers is presented in Fig. 13(b). The morphology of HDF cells was further analyzed (Fig. 13(d)). On day 1 after cell culture, cells exhibited a rounded shape across all samples. After 5 days, cells on the PVA nanofibers still retained their rounded shape. However, a slight elongation was observed in the PNP-2, and notably, the cells in the PNPC exhibited a greater degree of elongation compared to the other samples. Considering the previously mentioned results, we can conclude that the developed PNP-2 and PNPC nanofibers hold promise for use in wound healing, tissue engineering, or coating biomedical devices intended for human use.

## Conclusion

This study successfully synthesized ND@PDA nanoparticles by coating PDA on the surface of ND. We incorporated the synthesized ND@PDA into PVA to produce PNP nanofibers. This platform demonstrated excellent temperature elevation (97.1 °C) within 5 min at 808 nm. The ND@PDA within the nanofibers specifically absorbed the 808 nm NIR light during irradiation, generating heat that led to a rapid temperature increase. Additionally, it exhibited strong light absorption and generated singlet oxygen free radicals upon NIR irradiation. We observed antibacterial effects against *E. coli* and MRSA both with and without NIR exposure, achieving full antibacterial effects after irradiation. The elevated temperature and ROS killed the bacteria and disrupted the bacterial membrane within the biofilm, leading to its removal. Additionally, the PNPC nanofibers showed a significant increase in curcumin release within 30 min under NIR irradiation compared to without irradiation, followed by sustained release within the next 96 h. Furthermore, the PNP nanofibers showed no significant cytotoxicity and exhibited favorable biocompatibility due to the catechol groups’ enhanced cell adhesion. Hence, these results suggest that the developed nanofiber platform has versatile potential applications in the fields of medicine and biotechnology. However, in vitro studies do not fully reflect real biological environments, so future research should assess the performance of PNP nanofibers more accurately using in vivo biofilm models or chronic wound infection models. Such additional studies will play a crucial role in enhancing the clinical applicability of the developed nanofiber platform. We have included the comparative literature on application and properties of ND and PDA related scaffolds in Table 1.

**Table 1:** This study compares previous research NIR effect and antibacterial activity of ND and PDA-related composites

Formulations	Wavelength (nm)	Time (min)	Temp (°C)	Type of bacteria	Advantage	Limitations	Reference
N-carboxyethyl chitosan-Oxidized hyaluronic acid-ND	808	7	70	*S. aureus*, *E. coli*	Antibacterial, Wound healing, Cancer therapy	Excessive amounts of ND (CEC-OHA-1000) destroy the porous network structure; Antibacterial rate of CEC-OHA-250 against *E. coli* is lower compared to other concentrations	[62]
Carboxylated ND, Poly-aminated ND	808	7	~ 50	*S. aureus*, *E. coli*	Antibacterial, Tumor cell ablation	Minimum bactericidal concentration of ND is high; only 40% of cancer cells are eliminated	[63]
Porphyrins-Detonation ND@ Silver nanoparticles	415	30	-	*S. aureus*	Antibacterial, Biofilm suppression	Biocompatibility not shown; irradiation time is very long for bacteria killing	[64]
PDA nanospheres-6-carboxyfluorescein-terminated *S. aureus*-binding aptamer	808	3	76.9	*S. aureus*	Antibacterial, Biofilm suppression	Biocompatibility study required	[65]
PDA/ Poly(L)-lactide	-	30	~ 45	*S. aureus*, *E. coli*	Antibacterial	Antimicrobial effect decreases as PDA content increases, low temperature increase rate	[66]
PVA/ND@PDA-2%	808	5	97.1	MRSA, *E. coli*	Antibacterial, Biofilm removal, Wound healing		This study

*Staphylococcus aureus*: *S. aureus*, *Escherichia coli*: *E. coli*; Nanodiamond: ND; Polydopamine: PDA; Polyvinyl alcohol: PVA

## Electronic supplementary material

Below is the link to the electronic supplementary material.

Supplementary Material 1

## Data Availability

No datasets were generated or analysed during the current study.
