# Supplementary material for: NIR-activated catechol-functionalized nanodiamond nanofibers for accelerating on-demand MRSA and E. coli biofilm eradication
Source: J Biol Eng. 2025 Feb 5;19:2. doi: 10.1186/s13036-024-00469-6 (PMC11796056; doi:10.1186/s13036-024-00469-6)
Supplement: Supplementary file 1 — Supplementary Material 1 [file 13036_2024_469_MOESM1_ESM.docx]

**Supporting information**

**NIR-activated catechol-functionalized nanodiamond nanofibers for accelerating on-demand MRSA and E. coli biofilm eradication**

Hyeonseo Park^1,2+,^ Tejal V. Patil^1,2+^, Jieun Lee^1,2^, Hojin Kim^1,2^, Ki-Taek Lim^1,2,3*^

*^1^Department of Biosystems Engineering, Kangwon National University, Chuncheon-24341, Gangwon-do, Republic of Korea.*

*^2^Interdisciplinary Program in Smart Agriculture, Kangwon National University, Chuncheon-24341, Gangwon-do, Republic of Korea.*

*^3^Institute of Forest Science, Kangwon National University, Chuncheon-24341, Gangwon-do, Republic of Korea.*

^+^ Equally contributed

***Corresponding Author:** ktlim@kangwon.ac.kr


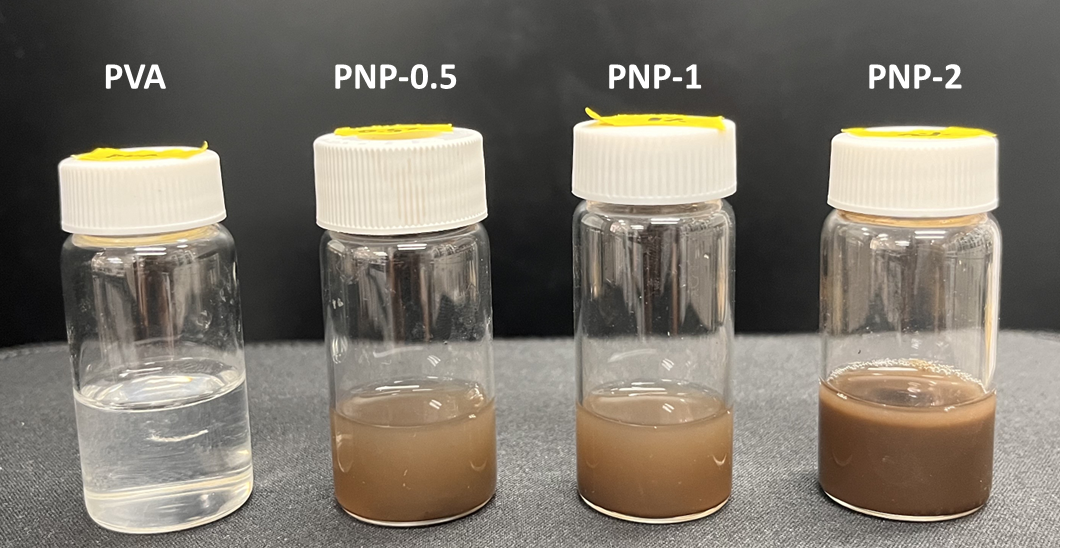


**Figure S1**. Pictures of PVA and 0.5, 1, 2% concentrations of PVA/ND@PDA solutions.


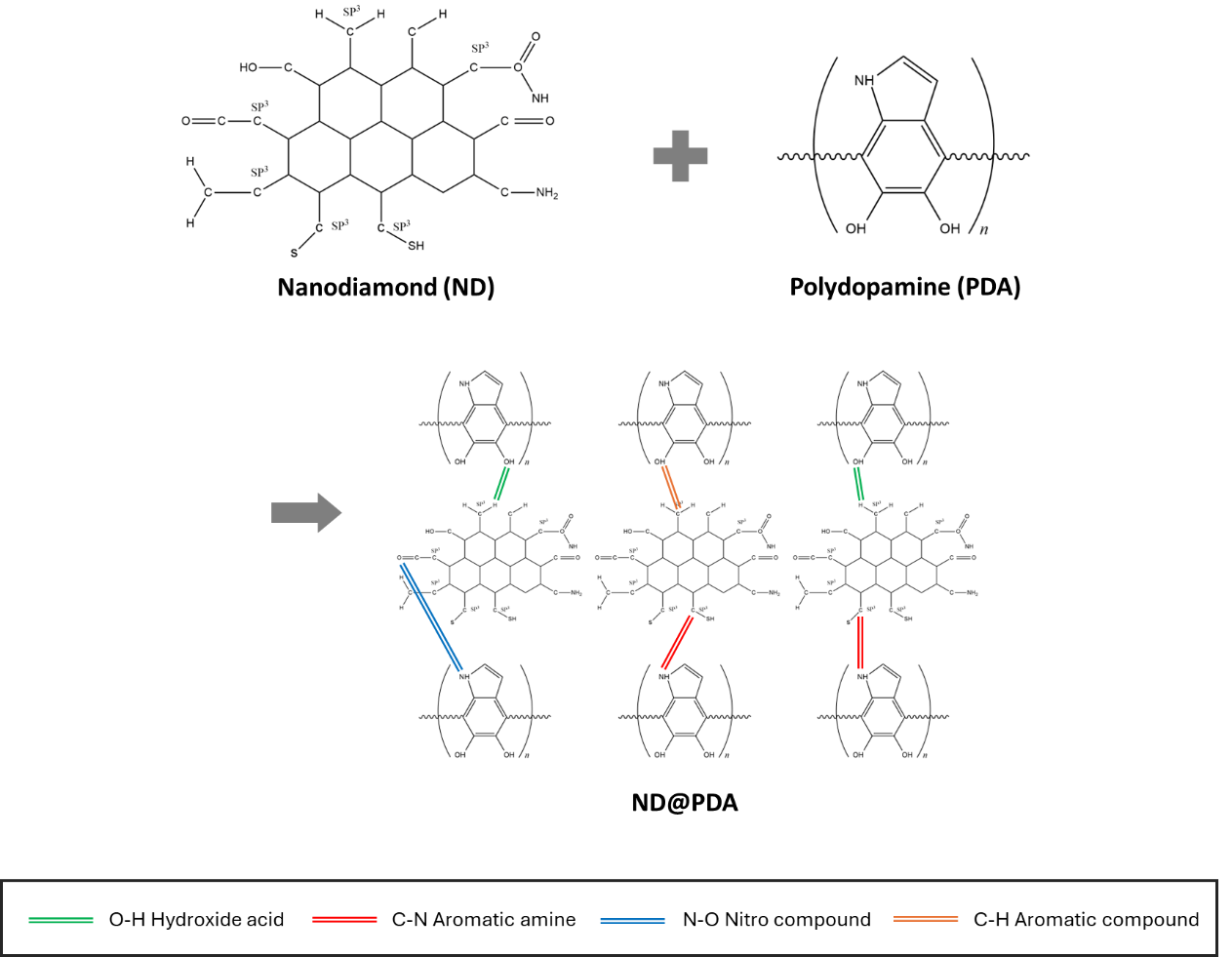


**Figure S2**. Chemical structure and interactions for surface coating of ND using PDA.

**
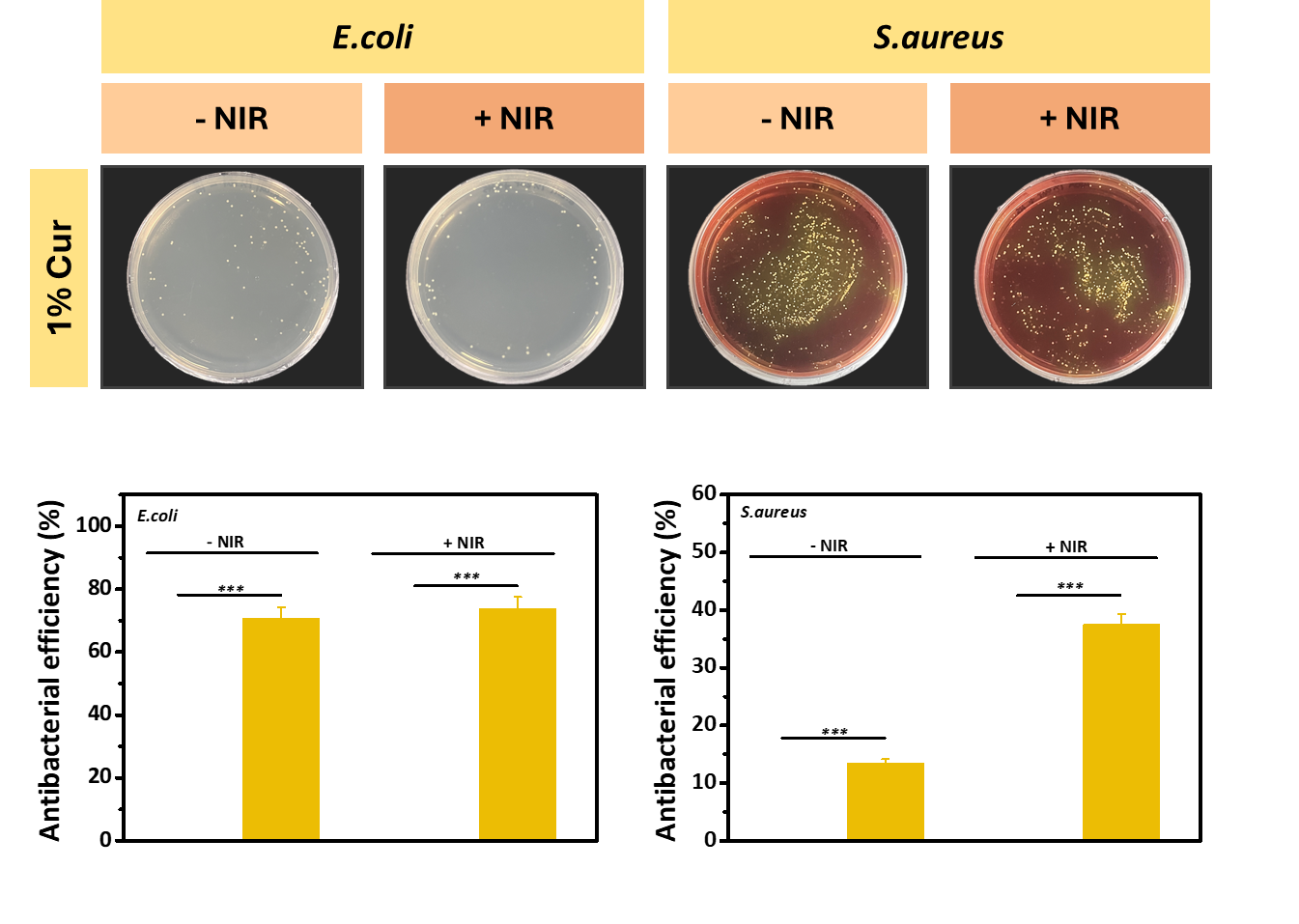
**

**Figure S3**. Photos of *Escherichia coli* and Methicillin-resistant *Staphylococcus aureus* bacterial colonies incubated for 6 h and their antibacterial efficiency percentage of 1% curcumin. Data are statistically significant at mean ± SD, not significant - ns, *p < 0.05, **p < 0.01, *** p < 0.001 in the triplicate experiment.


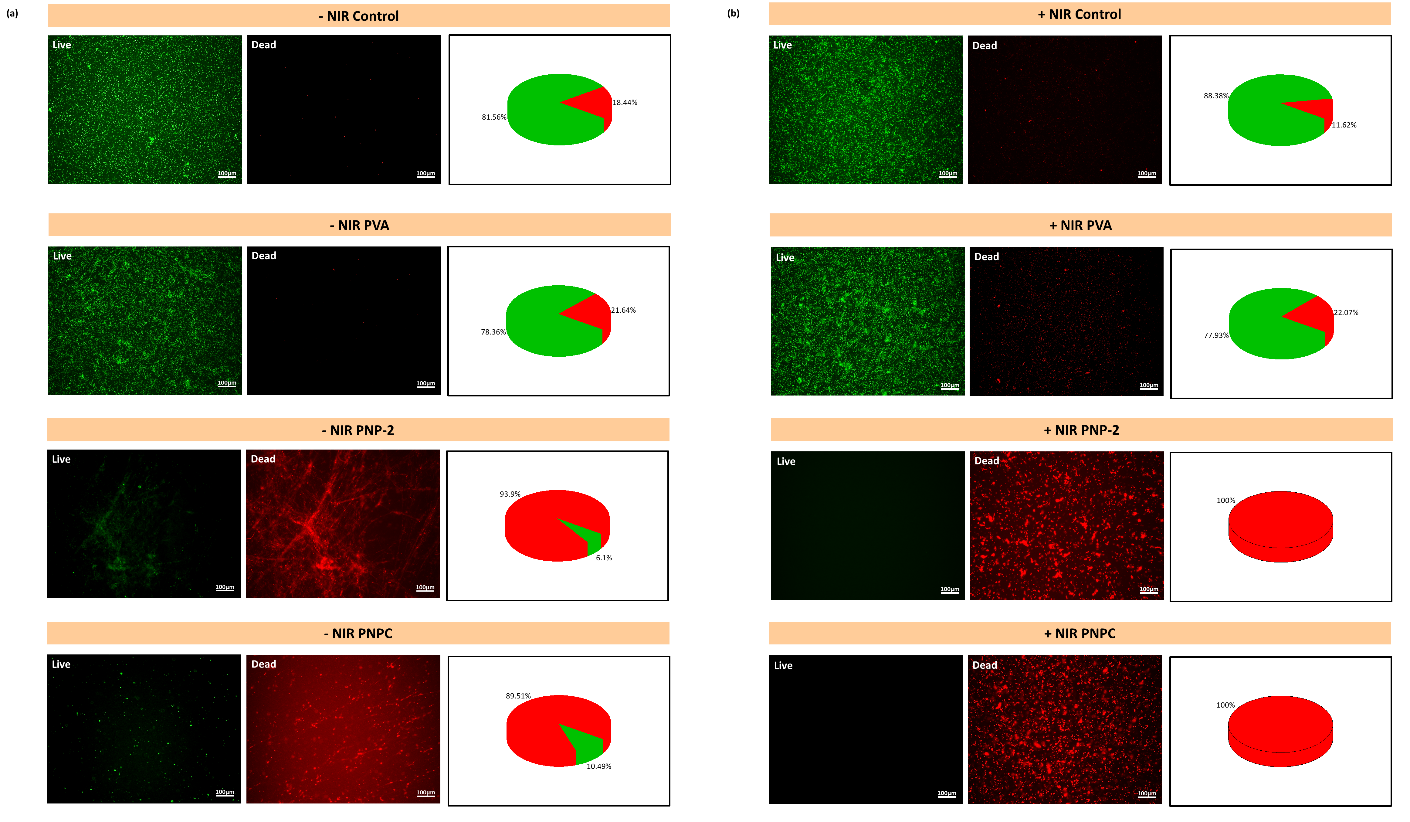


**Figure S4**. Live, dead images of *Escherichia coli* and quantitative analysis graph (a) without and (b) with irradiation of 1.0W/cm^2^ NIR.

**
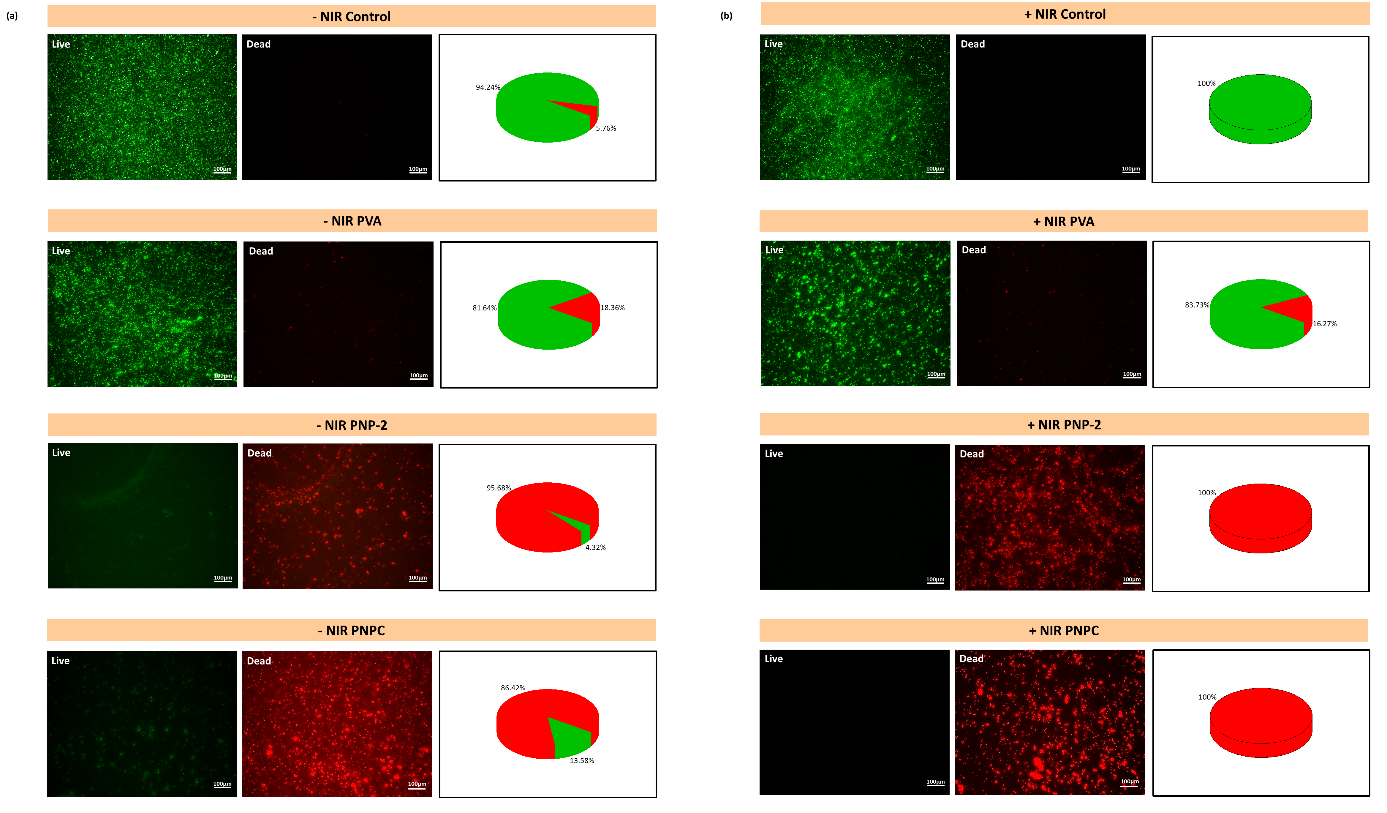
**

**Figure S5**. Live, dead images of Methicillin-resistant *Staphylococcus aureus* and quantitative analysis graph (a) without and (b) with irradiation of 1.0W/cm^2^ NIR.
